# Therapeutic effects of a novel electrode for transcranial direct current stimulation in ischemic stroke mice

**DOI:** 10.7150/thno.90779

**Published:** 2024-01-21

**Authors:** Da Hee Jung, Jae Ho Lee, Hong Ju Lee, Jang Woo Park, Young-Jin Jung, Hwa Kyoung Shin, Byung Tae Choi

**Affiliations:** 1 Department of Korean Medical Science, School of Korean Medicine, Pusan National University, Yangsan 50612, Republic of Korea.; 2 Graduate Training Program of Korean Medical Therapeutics for Healthy Aging, Pusan National University, Yangsan 50612, Republic of Korea.; 3 Korea Radioisotope Center for Pharmaceuticals, Korea Institute of Radiological & Medical Sciences, Seoul 01812, Republic of Korea.; 4 School of Healthcare and Biomedical Engineering, Chonnam National University, Yeosu 59626, Republic of Korea.

**Keywords:** Transcranial direct current stimulation, High-definition electrode, Stroke, RNA sequencing, Sterol regulatory element-binding protein

## Abstract

**Rationale:** Non-invasive transcranial direct current stimulation (tDCS), a promising stimulation tool to modulate a wide range of brain disorders, has major limitations, such as poor cortical stimulation intensity and focality. We designed a novel electrode for tDCS by conjugating a needle to a conventional ring-based high-definition (HD) electrode to enhance cortical stimulation efficacy.

**Method:** HD-tDCS (43 µA/mm^2^, charge density 51.6 kC/m^2^, 20 min) was administered to male C57BL/6J mice subjected to early-stage ischemic stroke. Behavioral tests were employed to determine the therapeutic effects, and the underlying mechanisms of HD-tDCS were determined by performing RNA sequencing and other biomedical analyses.

**Results:** The new HD-tDCS application, showing a higher electric potential and spatial focality based on computational modeling, demonstrated better therapeutic effects than conventional HD-tDCS in alleviating motor and cognitive deficits, with a decrease in infarct volume and inflammatory response. We assessed different electrode configurations in the new HD electrode; the configurations variously showed potent therapeutic effects, ameliorating neuronal death in the peri-infarct region via N-methyl-D-aspartate-dependent sterol regulatory element-binding protein 1 signaling and related inflammatory factors, further alleviating motor and cognitive deficits in stroke.

**Conclusion:** This new HD-tDCS application showed better therapeutic effects than those with conventional HD-tDCS in early-stage stroke via the amelioration of neuronal death in the penumbra. It may be applied in the early stages of stroke to alleviate neurological impairment.

## Introduction

Transcranial direct current stimulation (tDCS) applies a weak level of direct current to the scalp over a target cortical area to modulate neuronal excitability 1, 2. tDCS is applied using a non-invasive cortical device; it is a safe and well-tolerated treatment with no potentially harmful side effects. It is a promising stimulation tool and may help modulate a wide range of brain disorders 2-4. Stroke survivors have varying permanent disabilities and public health problems because most of them never fully recover from post-stroke deficits 5, 6. Due to the varying degrees of motor and neuropsychological disabilities that they experience, they require physical and cognitive rehabilitation 7.

Treatment using tDCS can effectively enhance neurorehabilitation, including motor and cognitive functions, for the long-term recovery of patients with neurological disorders 8, 9. Therefore, the therapeutic effects of tDCS are considered a potential neuromodulatory apparatus for patients with stroke to facilitate their functional recovery from motor and cognitive deficits 8, 10, 11. However, tDCS presents major concerns because of variations in individual patient characteristics, as well as inconsistent findings due to its poor stimulation focality and efficacy on cortical excitability, as reported in clinical trials 12-14.

The electrode characteristics in tDCS therapy are decisive because their size and shape determine the current density, location, and spatial distribution at target sites 15-17. Traditional tDCS employs large sponge-based rectangular pads that stimulate non-focal diffusion of current because of the large scalp area 12, 13, 18. To overcome this specificity limitation of a traditional tDCS electrode, a new type of smaller ring-based high-definition (HD) electrode has been employed to provide more targeted current delivery and spatial focality based on multiple electrodes 19, 20. Furthermore, concentric electrodes, as alternative versions of the HD-tDCS montage, have been proposed for better control of current distribution 21.

To improve the spatial focality and efficacy on cortical excitability of tDCS, we designed a new electrode by conjugating a needle to a conventional ring-based small HD electrode, as the delivery of current mainly depends on the geometry of the electrodes 16, 22. The electrode configuration of tDCS produces different clinical outcomes depending on the stroke stage; therefore, treatment time should be carefully determined based on the injury mechanisms of cerebral ischemia 11, 23, 24. However, most tDCS treatments have been focused on rehabilitation of chronic stroke disorders based on interhemispheric competition models, such as anodal tDCS application over the lesional hemisphere or cathodal tDCS over the contralesional hemisphere 11, 25. Previous research has demonstrated that during the subacute stage of ischemic stroke, anodal tDCS over the contralesional cortex promotes better recovery of neurological function compared with other configurations 26.

Firstly, we compared the predicted electric fields of the cortex and spatial focality of the newly designed HD electrode with those of the conventional electrode through computational modeling. Then, we applied these electrodes to mice in the early stage of ischemic stroke to determine whether this treatment prevents cell death and inflammation, which are the main ischemic injuries during this period, further leading to better functional recovery after stroke insults 27, 28. This study aimed to compare the therapeutic efficacy of the new HD-tDCS application with that of conventional tDCS through behavioral assessments involving motor and cognitive functions and to identify the potential therapeutic effects of the new HD-tDCS application by the underlying mechanism of neuroprotection from stroke insults.

## Methods

### Experimental procedures

Six-week-old male C57BL/6J mice (weight, 19-22 g) were obtained from Hana Biotech (Gyeonggi-do, Republic of Korea). A total of 94 mice were included and randomly divided into different experimental groups. We calculated a minimized animal sample size using G*Power 3.1 software (Heinrich-Heine-Universität, Düsseldorf, Germany, http://www.gpower.hhu.de/). The group sample size was calculated based on the latency of the pole test from our previous results (n=6/group, effect size f=0.8, α=0.05, and β=0.2). Consequently, group sample sizes of n>4 were used for the behavioral tests. HD-tDCS (43 µA/mm^2^, charge density 51.6 kC/m^2^, 20 min) was administered over the motor cortex for 5 consecutive days, from 3 to 7 days after middle cerebral artery occlusion (MCAO).

First, we performed computational modeling to compare the predicted current fields and spatial focality of the new HD electrode on the cortex with those of the conventional electrode. Next, the anode HD electrode was placed over the motor cortex of the brain in an ischemic stroke model to compare the therapeutic efficacies of the conventional and new HD electrodes. Mice were divided into three groups (n=5/group) to undergo transient MCAO, MCAO+tDCS-F (conventional ring-based flat HD anode electrode positioned over the contralesional cortex and referenced over the skin of the neck as an extracephalic one), and MCAO+tDCS-FN (new needle-conjugated ring-based anode HD electrode positioned over the contralesional cortex). Third, to determine the therapeutic efficacy of the new HD electrode according to different electrode configurations, five groups (n=5-6/group) were included. These five groups were as follows: control, MCAO, MCAO+sham (MCAO surgery with sham stimulation), MCAO+tDCS-FN(cA) (new anode HD electrode positioned over the contralesional cortex), and MCAO+tDCS-FN(iC-cA) (new anode HD electrode positioned over the contralesional cortex and cathode over the ipsilesional cortex). Fourth, we included the following three groups (n=3/group) to analyze possible neuroprotective genes involved in the therapeutic effect by RNA sequencing (RNA-seq) analysis (Ebiogen, Seoul, Republic of Korea): MCAO, MCAO+tDCS-FN(cA), and MCAO+tDCS-FN(iC-cA). Finally, further biomedical analyses were performed to verify the potential signaling for the therapeutic effects of the new HD electrode in the five groups (n=5-6/group).

All behavioral tests for motor and cognitive functions were performed from 7 to 10 days after MCAO, and the mice were sacrificed on day 10, as defined in the experimental schedule. All samples were subjected to quantitative real-time polymerase chain reaction (qPCR), western blot, and immunofluorescence analyses. The peri-infarct region was used as an independent sample. All experiments were approved by the Pusan National University Animal Care and Use Committee for the Ethical Use of Animals (PNU-2022-0149).

### Animals

All 94 6-week-old male C57BL/6J mice obtained from Hana Biotech (Gyeonggi-do, Republic of Korea) had free access to food and water (22±1 °C, ventilated and maintained at a 12-h light-dark cycle). Mice were randomly divided into different groups and allocated in a blinded manner.

### Three-dimensional HD-tDCS simulation

For 3D HD-tDCS simulation, magnetic resonance (MR) and micro-computed tomography (micro-CT) images were acquired from the head and neck of C57BL/6 mice. The MR image was obtained using a 9.4-T MRI scanner (Biospec 94/20 usr, Bruker, Germany). Anatomical MR images were acquired using a T2-weighted 3D TurboRARE sequence with the following parameters: repetition time, 2500 ms; echo time, 56 ms; field of view, 20×20×20 mm; matrix size, 256×256×128; rare factor 8, echo spacing, 14 ms; and number of excitations, 1. Micro-CT images were acquired using a SkyScan 1278 (Bruker). The imaging parameters were as follows: voltage, 70 kV; current, 200 uA; resolution, 9.982 um; exposure time, 473 ms; rotation step, 0.2°; filter, Al 1 mm; and trajectory, 360°. The acquired micro-CT images were reconstructed using the SkyScan NRecon software (ver. 1.7.4.6). A 3D mouse anatomical head model (skin, bone, and brain) was extracted using MRI and micro-CT scans. The skull mesh model was extracted from the micro-CT images, and the skin and brain mesh models were extracted using the MRI scans and itk-SNAP (itk-SNAP v3.8.0, www.itksnap.org). The boundary element model solver with the MoM algorithm used three mesh models (conductivity properties: scalp, 0.465; skull, 0.015; cerebrospinal fluid, 1.65; and brain, 0.3) for electric field analysis. The graphic user interface SW for the tDCS simulation using the MoM algorithm was implemented using MATLAB 2022b (MathWorks, Natick, MA).

### Focal cerebral ischemic model

Transient focal cerebral ischemia was induced using MCAO/reperfusion, with the intraluminal filament technique. Deep anesthesia in mice was achieved by administering facemask-delivered isoflurane at a concentration of 2% for induction and 1.5% for maintenance, along with a mixture of 80% N_2_O and 20% O_2_. The absence of cardiovascular changes upon administering a tail pinch was used as a measure of the depth of anesthesia. Throughout the procedure and reperfusion, a fiberoptic probe was attached to the exposed skull over the left middle cerebral artery. This enabled continuous monitoring of regional cerebral blood flow (CBF) using the PeriFlux Laser Doppler System 5000 (Perimed, Stockholm, Sweden). Following distal ligation of the left external carotid artery, a 7-0 silicon-coated monofilament (Duccol, Sharon, MA, USA) was inserted into the ventral incision on the common carotid artery and then advanced through the internal carotid artery to obstruct the left middle cerebral artery and induce MCAO. The removal of the filament after 40 min to allow for reperfusion was confirmed using a laser Doppler flowmeter (Perimed). Following MCAO induction, all included animals experienced a minimum of 80% CBF reduction from baseline. Prior to reperfusion, exclusion criteria were set at a maximum of 30% CBF reduction from baseline. Throughout the procedure, the mouse's body temperature was maintained at 37.5°C using a Panlab thermostatic heating blanket (Harvard Apparatus, Holliston, MA, USA). Each mouse was returned to its cage to recover after the surgery. Moistened food pellets were provided in the home cages throughout the study to ensure eating and hydration. The welfare, health, and activity of the mice were evaluated by weighing them once a day and observing them twice a day after the surgery. If a mouse's weight decreased by more than 20%, it was euthanized.

### High-definition transcranial direct current stimulation

**Figure 1:**
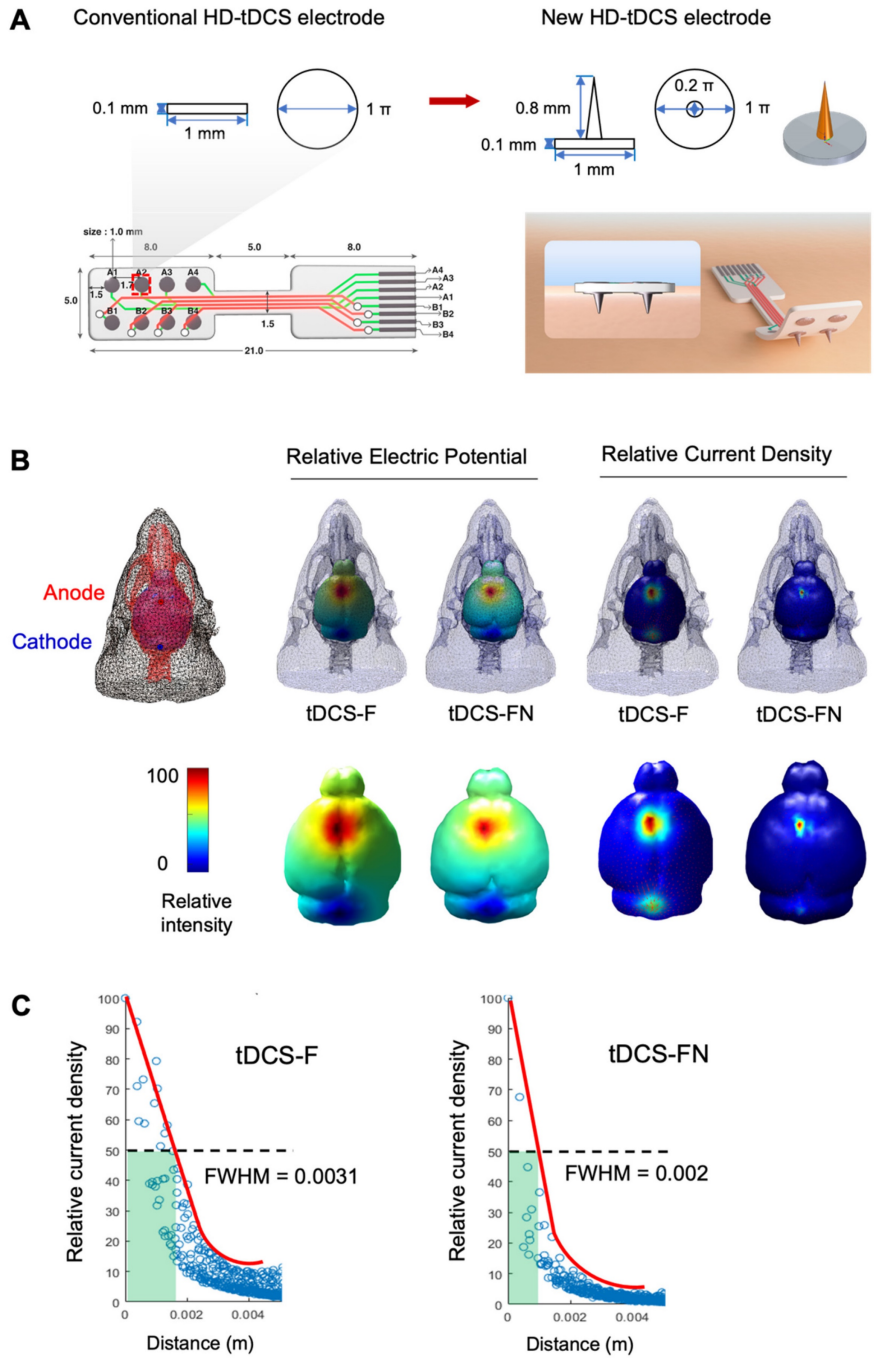
** Comparison of electric fields between conventional and new HD-tDCS applications in stroke. (A)** Schematic diagram of the conventional ring-based HD electrode and the new HD electrode. **(B)** 3D simulation of HD-tDCS. Higher relative electric potential and current density were induced in the new HD electrode compared to those with the conventional HD electrode. The maximum value is 100%, and the minimum value is 0%.** (C)** The FWHM value of HD-tDCS. The area representing the FWHM value from the peak value of current density under the electrode was smaller with the new HD electrode than that with the conventional electrode. HD-tDCS, high-definition transcranial direct current stimulation; F, conventional electrode; FN, new needle-conjugated electrode; FWHM, full width at half maximum.

Both the conventional ring-based HD and the new HD electrodes were employed for HD-tDCS. The new HD electrode has a greater surface area than the conventional one (1 mm^2^ vs. 0.785 mm^2^) despite both electrodes having the circular shapes and radius (0.5 mm) (**Figure 1A**). The electrodes were positioned over the motor cortex (1.25 mm left and right, +1.2 mm from the bregma) and treatment with HD-tDCS current was applied continuously for 20 min (43 µA/mm^2^, at a charge density of 51.6 kC/m^2^) using 8CH-tDCS (Neuro Rehap, Busan, Republic of Korea) under 2% isoflurane (Choongwae) on a heating pad (37℃). The reference position was placed over the skin of the neck as an extracephalic one using a needle (0.25 mm diameter, 40 mm long). Two different electrode configurations were used: anode contralesional stimulation (cA) for tests between conventional and new electrodes, and cA and bi-hemispheric stimulation (iC-cA) for tests of new electrode based on configuration. The non-HD-tDCS-treated groups were anesthetized for 20 min. HD-tDCS was applied once a day from 3 to 7 days after stroke for 5 consecutive days. To prevent the stimulation break effect, the current intensity was ramped up for 30 s, while no current was applied in the sham group. The HD-tDCS intervention was performed at approximately the same time (10:00 am). Pain or stress resulting from the HD-tDCS application was estimated by measuring the weight.

### Behavioral tests

The behavioral tests were conducted in a quiet room (light intensity <50 lx, temperature 22±1°C), and all animals were acclimated in the test rooms for over 30 min. All behavioral tests were performed by blinded observers and independent researchers.

Corner test: Two vertical boards (30×20 cm) were attached at an angle of 30°. Ten trials were performed for each mouse. The mouse was stimulated to fully rear up along either board, then permitted to return to the open end; this was recorded. The number of ipsilateral turns was calculated as a percentage.

Pole test: The mice were placed on the tip of a wooden pole (1×50 cm) with their heads upward. The total latency, including the time to turn the head down and to descend toward the floor, was measured in five trials and calculated by averaging three recordings for each mouse. The subsequent attempts were limited to a maximum duration of 120 s.

Open-field test (OFT): The open field (30×30×50 cm^3^) was a white square box divided into a center (15×15 cm^2^) and surrounding zone. After habituation for 5 min, test sessions took place in which mice freely explored the environment for 15 min, and were monitored using a SMART v3.0 video tracking system (Panlab, Barcelona, Spain). The total distance, mean speed, and time spent in the central zone were analyzed.

Novel object recognition (NOR) test: The mice were exposed to three successive trials in an inter-trial interval of 24 h in an open-field box (60×60×30 cm^3^). After being allowed to explore the box for 10 min, the mice were placed in a chamber containing two copies of a single object (4×4×11 cm^3^ round bottles positioned 10 cm from the wall, referred to as “A” and “A*,” respectively). The total time spent exploring each of the two objects was measured using a SMART v3.0 video tracking system (Panlab). Following a delayed period of 24 h for the test phase, one of the objects was exchanged for a highly dissimilar object (a 4.5×5.5×9 cm^3^ square bottle, referred to as B). The recognition index measured the time spent exploring the novel object (B) for 10 min. The recognition index (%) was calculated using the following equation: recognition index (%) = B/(B + A)×100.

Trace fear conditioning: Mice were fear-conditioned in standard operant chambers (Panlab) located inside sound-attenuating boxes in an isolated testing room. The experiment was conducted over a period of 3 days. On the day of habituation, mice were familiarized with the context for 10 min. On the fear conditioning day, mice were placed in context A for 3 min and subjected to trace fear conditioning in four trials. The mice sequentially received 10 s of pure tones (2.8 kHz, 85, 0.5, and 1 Hz) and white noise for 10 s (0.5 s duration at 1 Hz, 85 dB) as the conditioned stimuli, and an 18-s gap (trace interval) followed by foot shock (1 s, 0.3 mA). The chamber was cleaned using 70% ethanol at the end of each trial. On the test day, the animals were returned to context A for 6 min under the same conditions as those on the fear conditioning day for a contextual fear recall test. After 2 h, the mice were placed in a new context B for the cue recall test, during which the mice received a conditioned stimulus for 20 s in 80-s intervals during the four trials, after habituation for 3 min. Freezing behavior was used as an index of fear and was measured automatically using the FREEZING software program, version 2.0.04 (Panlab).

### Tissue preparation

For the anatomical studies, the mice were anesthetized and transcardially perfused with phosphate-buffered saline (PBS, pH 7.4) followed by 4% paraformaldehyde (PFA) in PBS. The brain and scalp were extracted and post-fixed in 4% PFA/PBS at 4°C for 24 h before being cryoprotected in 30% sucrose. The brains were stored at -80 °C and embedded in an optimal cutting temperature medium. For the molecular studies, the peri-infarct region of the cortex was extracted and immediately stored in liquid nitrogen. Nissl staining was performed from 1.1 mm to -1.9 mm from the bregma, and immunofluorescence staining was performed from 1.1 mm to 0.02 mm from the bregma. Other molecular analyses were conducted by obtaining the peri-infarct region from 1.1 mm to 0.1 mm from the bregma.

### Hematoxylin and eosin (H&E) staining

The brain and scalps of mice were cryosectioned for 25 µm and 5 µm, respectively and stained with Harris hematoxylin solution (Cancer Diagnostics, Durham, NC) for 5 min, then rinsed with tap water. Next, we progressed to the differentiation step with 1% HCl, and the bluing step with an alkaline solution. Finally, staining was performed with eosin Y alcoholic solution (Cancer Diagnostics). We covered H&E-stained sections with mounting medium (ZC0123; Vector Laboratories, Burlingame, CA, USA) and photographed them using a Zeiss Axio Imager A1 microscope (Zeiss, Germany).

### Measurement of infarct area

The collected serial sections of the whole brain were cryosectioned to acquire 25 µm at intervals of 75 µm by a CM3050 cryostat (Leica Microsystems, Wetzlar, Germany). All sections were stained with Nissl and examined to calculate the infarct area. Tissues were dehydrated with 50, 70, 80, 90, and 100% ethanol and stained with 0.1% fast cresyl violet acetate (C5042, Sigma-Aldrich) for 5 min. They were then washed in double-distilled water five times and covered with mounting medium (ZC0123; Vector Laboratories). As Nissl staining can clearly identify the infarct area at 1.1 mm to -1.9 mm from the bregma using the mouse brain atlas, Nissl-stained sections were photographed using a Zeiss Axio Scan. Z1 Slide Scanner (Axio Scan Z1; Zeiss). The infarct area was automatically drawn using Image-J v1.53 software (NIH, Bethesda, MD) and calculated using the following equation: corrected infarct area = [contralateral hemisphere area - (ipsilateral hemisphere area - infarct area)] × thickness.

### RNA sequencing

Total RNA was extracted from the peri-infarct region of the cortex using TRIzol reagent (Invitrogen, Carlsbad, CA, USA). Quality control of the raw sequencing data was performed using FastQC software. The total library was constructed using the Quant Seq 3′ mRNA-Seq Library Prep Kit FWD (Lexogen, Vienna, Austria). High-throughput single-end 75 sequencing was performed using NextSeq 500/550 (Illumina, San Diego, CA). During differentially expressed genes (DEG) analysis, gene sets exhibiting a fold change of >1.50, normalized data (log_2_) >2, and a *P*-value of <0.05 (indicating the level of statistical significance). Furthermore, DEG and Gene Ontology (GO) analyses were conducted using an Excel-based DEG analysis (ExDEGA) software package (Ebiogen Inc., Seoul, Republic of Korea). The Database for Annotation, Visualization, and Integrated Discovery (DAVID, https://david.ncifcrf.gov/) was used to analyze DEGs in Kyoto Encyclopedia of Genes and Genomes (KEGG) pathway enrichment and GO functional analyses. GO terms were considered significant when the false discovery rate adjusted *P*-value was <0.05. The Search Tool for the Retrieval of Interacting Genes/Proteins database was used, followed by Cytoscape software (version 3.5.1) for protein-protein interaction (PPI) network analysis. Marked modules were selected based on molecular complex detection (MCODE) with scores OF >2 in Cytoscape. The 10 hub genes identified were used to locate the protein clusters of highly interconnected regions within the initial network belonging to the same biological pathway.

### Quantitative real-time PCR

Total RNA was extracted from the peri-infarct region of the cortex using TRIzol reagent (Invitrogen). The concentration and RNA quality were determined (260/280 nm ratios) by a Nanodrop Spectrophotometer (nd-1000; Thermo Fisher Scientific, Waltham, MA). The desired absorbance ratio of 260/280 nm was 1.8-2.0. The total RNA (2 µg) for each sample was reverse transcripted to cDNA using a TOPscript™ cDNA Synthesis Kit (EZ005S; Enzynomics, Daejeon, Republic of Korea) in 20-µL reaction solutions. Then, 2 µg cDNA and gene-specific primers (25 pmol) were added to the SYBR green master mix (RR420A; Takara Bio, Kusatsu, Japan) and subjected to PCR amplification. The following primer sets were used:

*Insig1* forward, 5′-ACCTGGGAGAACCACACAAG -3′

*Insig1* reverse, 5′-CTTCGGGAACGATCAAATGT -3′

*Bag5* forward, 5′-TGGGAACCTGTCTGAGATCC -3′

*Bag5* reverse, 5′-GTCCGACTTCATGTCCAGGT-3′

*Egr2* forward, 5′-CGCCACACCAAGATCCAC-3′

*Egr2* reverse, 5′-AGCCCCCAGGACCAGAGG-3′

*Nfkb1* forward, 5′-CTGACCTGAGCCTTCTGGAC-3′

*Nfkb1* reverse, 5′-GCAGGCTATTGCTCATCACA-3′

*Bche* forward, 5′-GGGCAGTAAAGCATCCTGAG-3′

*Bche* reverse, 5′-GAGGGGAGAACGAACCTTTC-3′

*Hdac2* forward, 5′-AAAGGAGCAAAGAAGGCTAGG-3′

*Hdac2* reverse, 5′-GTCCTTGGATTTGTCTTCTTCC-3′

*Srit1* forward, 5′-TCGTGGAGACATTTTTAATCAGG-3′

*Srit1* reverse, 5′-GCTTCATGATGGCAAGTGG-3′

*U2af2* forward, 5′-GCACAGGAAGCGTAGTCACA-3′

*U2af2* reverse, 5′-GCCAGACCATCAGGAGTCAT-3′

*Gapdh* forward, 5′-GGGTGTGAACCACGAGAAAT-3′

*Gapdh* reverse, 5′-CCTTCCACAATGCCAAAGTT-3′

Cycling conditions for all samples comprised initial denaturation (95°C, 10 min), followed by denaturation (95°C, 10 s), annealing (60°C, 15 s), and melting curve analysis (72°C, 10 s) for 40 cycles.

Relative expression data were quantified using 2^-ΔΔCt^, where the cycle threshold (Ct) denotes the threshold value. Gene expression is indicated as the fold change in the control group.

### Enzyme-linked immunosorbent assay

The protein levels of high-sensitivity C-reactive protein (hs-CRP) and tumor necrosis factor-related apoptosis-inducing ligand (TRAIL) in the peri-infarct region were measured using mouse TRAIL (ab253210; Abcam, Cambridge, UK) and mouse hs-CRP ELISA kits (MBS026987; MyBioSource, San Diego, CA, USA). Tissue samples were dissected from the peri-infarct region. TRAIL ELISA samples were homogenized with PBS (10 mg tissue/0.1 mL PBS) and then centrifuged at 5,000 × *g* for 5 min at 4°C after two freeze-thaw cycles. hS-CRP ELISA samples in the tissue were homogenized with PBS and centrifuged at 1,000 × *g* for 10 min at 4°C. Standards and samples were applied to the titer plates. The final absorbance was measured at 450 nm using a SpectraMax 190 microplate reader (Molecular Devices, Sunnyvale, CA). Standard curves for recombinant TRAIL (2.458-600 pg/well) and hs-CRP (6.25-200 ng/well) were plotted for each plate. The average values of the samples were normalized to the total protein concentration.

### Western blotting

Frozen tissues from the peri-infarct region of the cortex were homogenized in a lysis buffer containing 250 mM NaCl, 5 mM EDTA (pH 7.5), 25 mM Tris-HCl (pH 7.5), 1% NP40, 1 mM phenylmethyl sulfonyl fluoride, 5 mM dithiothreitol, 0.1 mM sodium orthovanadate, 10 mM NaF, leupeptin, and protein inhibitor cocktail on ice. Tissue lysates were centrifuged at 13,000 rpm for 25 min at 4°C. Protein electrophoresis (20 µg) in 10% sodium dodecyl sulfate-polyacrylamide gel was performed, and protein was transferred to a nitrocellulose membrane (Whatman, Piscataway, NJ) using a blotting system (Bio-Rad, Hercules, CA). After blocking in a 5% solution of non-fat milk for 1 h at 20 °C, the membrane was incubated overnight at 4°C with the following primary antibodies: INSIG1 (1:100, ab70784, Abcam), BAG5 (1:500, sc-390832; Santa Cruz Biotechnology, Dallas, TX, USA), NFkB1 (1:500, 51-3500, Thermo Fisher Scientific), HDAC2 (1:500, sc-81599, Santa Cruz Biotechnology), U2AF2 (1:1000, MA5-35532, Thermo Fisher Scientific), SREBP1 (1:500, MA5-16124, Thermo Fisher Scientific), COX2 (1:500, 4842; Cell Signaling Technology, Danvers, MA, USA), NR2A (1:500, NBP2-22404; Novus Biologicals, Centennial, CO, USA), NR2B (1:500, NB100-74475, Novus Biologicals), pNR2B (1:200, 38-7000, Invitrogen), AKT (1:500, 38-7000, Invitrogen), pAKT (1:500, 4058, Cell Signaling Technology), phosphoinositide-3-kinase (PI3K; 1:500, 4257, Cell Signaling Technology), phosphorylated PI3K (pPI3K, 1:500, 4228, Cell Signaling Technology), and ß-actin (1:1000, A2066, Sigma-Aldrich). Primary antibodies were detected using horseradish peroxidase-coupled secondary antibodies (ADI-SAB-100 or -300; Enzo Life Sciences, Farmingdale, NY, USA) in an enhanced chemiluminescence system (Pierce, Rockford, IL, USA) using the Quant LAS-4000 imaging system (Fujifilm, Tokyo, Japan). Western blotting was performed using an IMT i-Solution v10.1 (Riverton, UT, USA). Protein levels/ß-actin ratios were calculated, and data are presented as percentages or mean values of the control group protein levels.

### Immunofluorescence

The peri-infarct region of the stroke-damaged brain was used for immunofluorescence analysis. Briefly, the brain sections were incubated with blocking buffer (1× PBS/7.5% house serum/0.3% Triton X-100) for 1 h at room temperature. They were then incubated overnight with the following primary antibodies (diluted in antibody dilution buffer, 1× PBS/1% bovine serum albumin/0.3% Triton X-100) at 4°C: ionized calcium binding adaptor molecule 1 (Iba1) (1:500, 019-19741; Wako Chemicals, Richmond, VA), CD68 (1:500, sc-135872, Santa Cruz Biotechnology), NeuN (1:500, MAB377, Millipore, Billerica, MA, USA), SREBP1 (1:100, NB100-74542, Novus Biologicals), pNFkB1 (1:100; PA5-37658, Invitrogen), and c-Cas3 (1:50; 9661, Cell Signaling Technology). The sections were washed with PBST and incubated with fluorescent secondary antibodies (A11001 or A11037; Invitrogen) for 2 h at room temperature. Sections were mounted using a mounting medium with DAPI (H-1200-10, Vector Laboratories), and images were captured using a fluorescence microscope (Zeiss Imager M1, Carl Zeiss) and fluorescence K1-fluo confocal microscope (#SMTH-0607-100T; NANOSCOPE Systems, Daejeon, Republic of Korea). Positive cells and integral optical density (IOD) were quantified in a blinded manner.

### Statistical analyses

Graphs were plotted and statistical analyses were conducted using SigmaPlot v12.5 (Systat Software, San Jose, CA, USA). All data were plotted as individual points with a mean±standard error of the mean. Normality tests were conducted for all one-way analyses using the Shapiro-Wilk and equal variance tests. When the samples did not satisfy the normality test, the chi-squared test was conducted, followed by the Kruskal-Wallis test. Statistical analyses were performed using one-way analysis of variance (ANOVA), followed by Tukey's post-hoc test. P<0.05 was considered a significant difference.

## Results

### Comparison of electric fields between conventional and new HD-tDCS applications

We performed computational modeling of the electric fields using the boundary element method to compare conventional and new HD-tDCS applications, as the current delivered to the cortex depends on the geometry of the electrodes (**Figure 1A**). The relative electric potential and current density were measured with the anode and cathode in the same position. The new HD-tDCS electrode resulted in a smaller stimulation area compared to that of the conventional electrode. For a three-dimensional (3D) HD-tDCS simulation of ten cortical areas, the predicted electric fields were mainly observed around the targeted electrode area. Moreover, tDCS with a needle-conjugated HD electrode showed a higher relative peak electric potential and current density of 12.9% (V/m) and 3.4% (C/m^2^), respectively, in the targeted cortex, compared with that generated with a conventional electrode (**Figure 1B**). For the further test of spatial focality, we calculated the full width at half maximum (FWHM) value based on the difference between the values of the two independent variables by calculating the relative current density according to geodesic distance (https://www.numerical-tours.com/matlab/fastmarching_6_sampling_surf/). The new HD-tDCS electrode (0.002 σ) resulted in a lower FWHM value than the conventional electrode (0.0031 σ), which created a narrower focal area of 64.5% (**Figure 1C**).

### Comparison of therapeutic effect between conventional and new HD-tDCS applications

**Figure 2:**
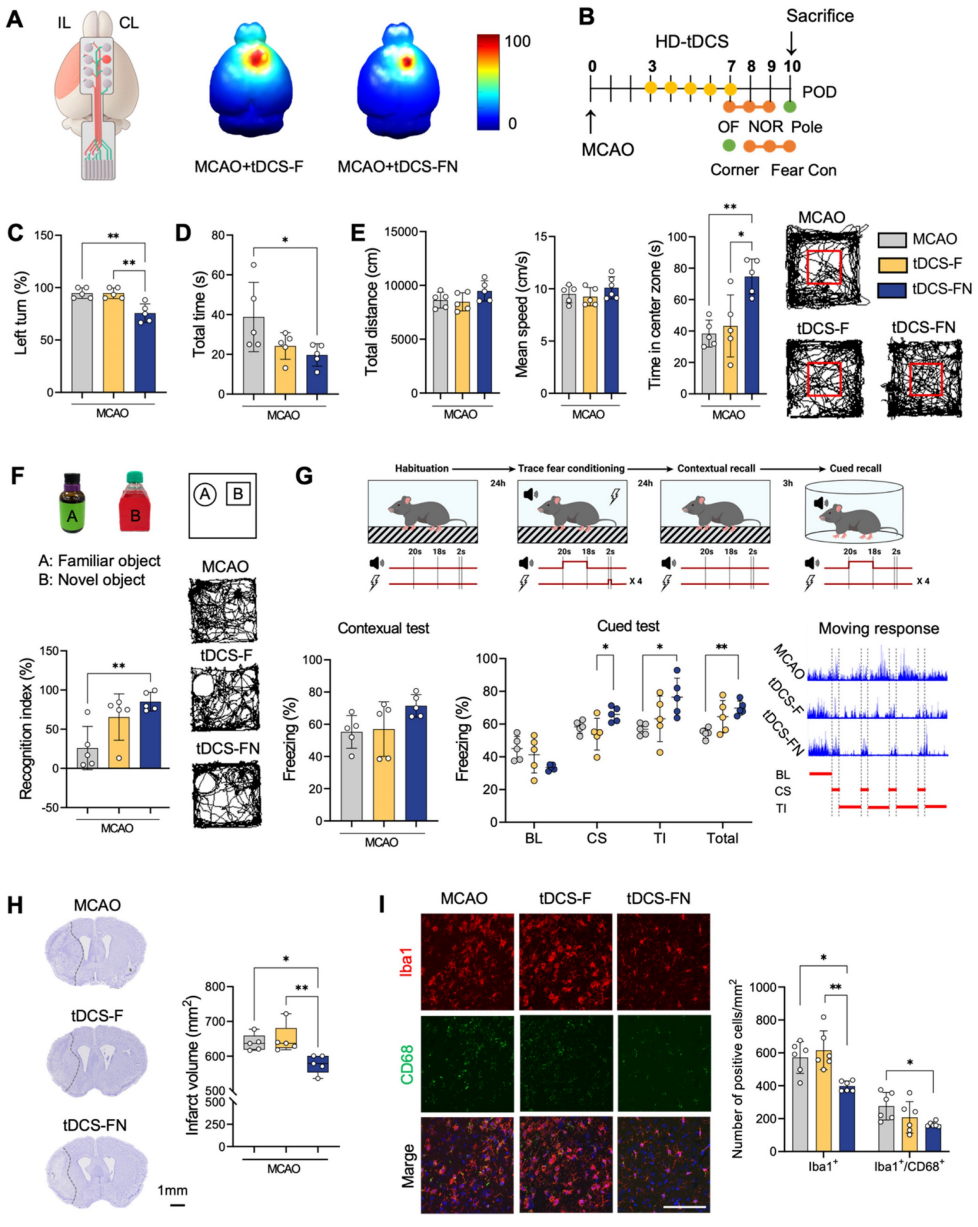
** Comparison of therapeutic effects between conventional and new HD-tDCS applications in stroke. (A)** Schematic diagram of the new HD electrode and 3D simulation of the HD-tDCS application. Higher relative electric potential and enhanced spatial focality were predicted in the motor cortex using the new HD-tDCS application compared with those using the conventional HD-tDCS application. **(B)** Timeline for HD-tDCS application and behavioral tests. Both HD-tDCS applications were used to treat the contralesional motor cortex once daily for 5 days. **(C)** Corner test: The percentage of left turns was significantly decreased in the MCAO+tDCS-FN group compared with that in the MCAO and MCAO+tDCS-F groups. **(D)** Pole test: The total time for the MCAO+tDCS-FN group was significantly decreased compared with that of the MCAO group. **(E)** Bar charts and performance images of the open-field test. The time in the center zone was significantly increased in the MCAO+tDCS-FN group compared with that in the MCAO and MCAO+tDCS-F groups. **(F)** Schematic subject images, bar charts, and performance images for the novel object recognition test. The recognition index was significantly enhanced in the MCAO+tDCS-FN group compared with that in the MCAO group. **(G)** Schematic diagram, bar charts, and schematic recording configuration of the movement during the fear conditioning. The percentage of freezing in the CS was significantly enhanced in the MCAO+tDCS-FN group compared with that in the MCAO+tDCS-F group.** (H)** Representative brain images (-0.1 mm from the bregma) and quantification of the relative infarct volume. Borders to the healthy tissue are indicated with a dot line. The infarct volume in 40 brain sections from 1.1 mm to -1.9 mm from the bregma was significantly reduced in the MCAO+tDCS-FN group compared with that in the MCAO+tDCS-F group. **(I)** Photomicrograph and bar chart showing the number of Iba1- and CD68-positive cells in the peri-infarct region. The number of Iba1-positive cells was significantly reduced in the MCAO+tDCS-FN group compared with that in the MCAO+tDCS-F group. All behavioral, morphological, and immunofluorescence analyses (n=5/group). Data are presented as means±SEMs. *P<0.05 and ***P<0.001 vs. each group using one-way analysis of variance with Tukey's test. IL, ipsilesional site; CL, contralesional site; MCAO, middle cerebral artery occlusion; HD-tDCS, high-definition transcranial direct current stimulation; F, conventional electrode; FN, new needle-conjugated electrode; BL, baseline; CS, cue stimulation; TI, trace interval. Magnification: ×400; scale bar=100 μm.

Similar to the computational modeling, the better relative electric potential and spatial focality of the new HD-tDCS application were predicted in the motor cortex compared to those with the conventional application (**Figure 2A and Figure S1A**). After HD-tDCS treatment over the contralesional motor cortex, as indicated in the experimental schedule (**Figure 2B**), we performed behavioral tests. Overall, the MCAO+tDCS-FN group, stimulated by the new needle-conjugated HD electrode, showed significant improvements in motor and cognitive functions compared with the conventional MCAO+tDCS-F group (**Figure 2C-G**). The MCAO+tDCS-F group showed less functional improvement than the MCAO group, although the difference was not significant. However, the MCAO+tDCS-FN group showed significant differences in all behavioral tests for motor function and cognition compared with the MCAO group. In addition, the MCAO+tDCS-FN group showed significant improvements in the behavioral test results, including those of the corner, open (time in center zone), and fear conditioning (cue stimulation) tests, compared with the MCAO+tDCS-F group (**Figure 2C-G**).

There were no histological injuries in the scalp or cerebral cortex of the HD-tDCS-treated groups compared with the non-treated or control group (**Figure S1B and C**). Prominent areas of the infarct region were detected in the striatum and cerebral cortex on the coronal brain section, along with additional regions such as the hippocampus and thalamus. Recovery of the infarct region by serial sectioning revealed a significantly lower infarct volume of 9.58% in the MCAO+tDCS-FN group compared to the MCAO groups (**Figure 2H and Figure S1D**). Immunofluorescence analysis of cluster of differentiation 68 (CD68) and ionized calcium binding adaptor molecule 1 (Iba1) as markers of microglial reactivity in the peri-infarct region showed a significantly decreased number of Iba1-positive cells in the MCAO+tDCS-FN group compared with that in the MCAO and MCAO+tDCS-F groups. Moreover, the number of Iba1/CD68-double positive cells was significantly reduced by the new HD-tDCS treatment compared with that in MCAO (**Figure 2I**). These results suggest that the new HD-tDCS treatment in the motor cortex alleviates motor and cognitive deficits by decreasing infarct volume and inflammatory response to ischemic stroke insults. Moreover, the new HD-tDCS application showed better therapeutic effects than the conventional application.

### Therapeutic effect of new HD-tDCS application according to electrode configuration

**Figure 3:**
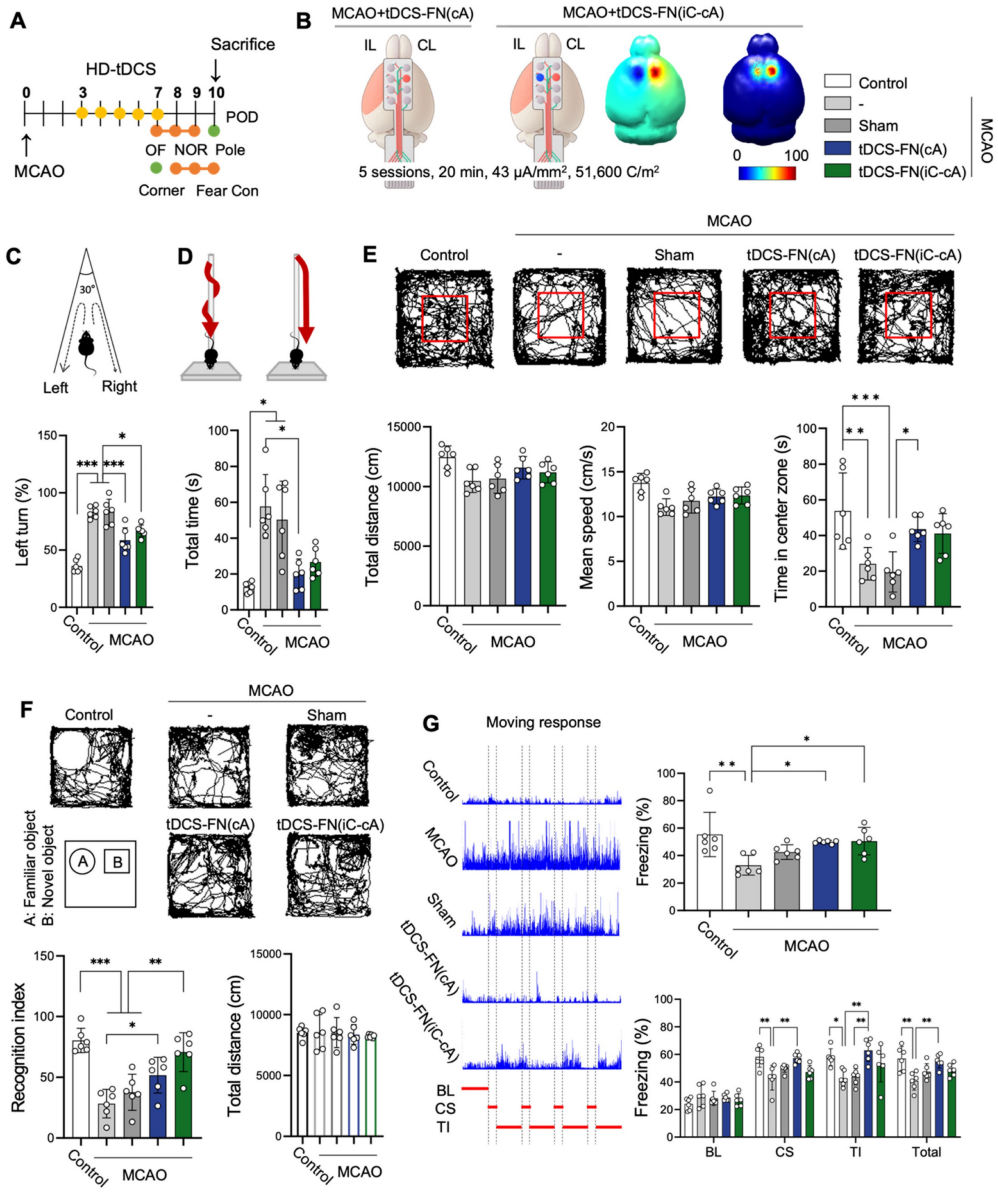
**Effect of the new HD-tDCS application according to electrode configuration in stroke. (A)** Timeline for HD-tDCS application and behavioral tests. **(B)** Schematic diagram of each electrode configuration and 3D HD-tDCS simulation, MCAO+tDCS-FN(cA), and bi-hemispheric MCAO+tDCS-FN(iC-cA). A higher relative electric potential was predicted on the targeted in new MCAO+tDCS-FN(cA) than bi-hemispheric stimulation. **(C)** Corner test: The percentage of left turns was significantly reduced following both HD-tDCS-FN treatments. **(D)** Pole test: The MCAO+tDCS-FN(cA) group demonstrated a marked decrease in the total time compared with that in the MCAO group. **(E)** Performance images and bar charts of the open-field test. The time in the center zone was significantly increased in the MCAO+tDCS-FN(cA) group compared with that of the MCAO group. **(F)** Performance images and bar charts of the NOR test. The recognition index was significantly enhanced in both the MCAO+tDCS-FN(cA) and MCAO+tDCS-FN(iC-cA) groups compared with that in the MCAO group. **(G)** Schematic recording of the configuration of movement and bar charts in the fear conditioning test. The percentage of freezing time was significantly increased in both the MCAO+tDCS-FN groups compared with that in the MCAO group in the contexture test, although with the CS, TI, and total of the cue test, it was enhanced only in the MCAO+tDCS-FN(cA) group. All behavioral analyses (n=6/group). Data are presented as means±SEMs. *P<0.05, **P<0.01, and ***P<0.001 vs. each group, using one-way analysis of variance with Tukey's test. IL, ipsilesional site; CL, contralesional site; MCAO, middle cerebral artery occlusion; HD-tDCS, high-definition transcranial direct current stimulation; FN, new needle-conjugated electrode; BL, baseline; CS, cue stimulation; TI, trace interval.

Because different electrode configurations produce specific neuronal excitability (for the brain activity), we employed two types of electrode configurations: MCAO+tDCS-FN(cA) (anode electrode over the contralesional site) and bi-hemispheric MCAO+tDCS-FN(iC-cA) (anode electrode positioned over the contralesional site and cathode over the ipsilesional site) (**Figure 3A and B**). For the computational modeling of electric fields, the MCAO+tDCS-FN(cA) group showed a higher relative peak electric potential and current density of 52.2% (V/m) and 7.2% (C/m^2^), respectively, at the targeted cortex, than the MCAO+tDCS-FN(iC-cA) group.

Both configurations of the new HD-tDCS application improved neurofunctional behaviors for motor function and cognition compared with MCAO. However, motor function was significantly improved in the MCAO+tDCS-FN(cA) group compared with that in the MCAO group in the corner, pole, and open (time in the center zone) tests, although MCAO+tDCS-FN(iC-cA) only improved in the corner test (**Figure 3C-E**). Both the MCAO+tDCS-FN(cA) and MCAO+tDCS-FN(iC-cA) groups showed a significant increase in the recognition index and percentage of freezing time compared with the MCAO group in the NOR and fear conditioning tests. However, the percentage of freezing, such as cue stimulation (CS), trace interval (TI), and total stages in the cue test significantly increased only in the MCAO+tDCS-FN(cA) group compared with the MCAO group (**Figure 3F and G**). These results suggest that both electrode configurations of the new HD-tDCS application alleviated motor and cognitive deficits in stroke; however, anodal treatment over the contralesional site presented better therapeutic effects than bi-hemispheric stimulation in the early stage of stroke.

### Transcriptomic analysis of new HD-tDCS application according to electrode configuration

**Figure 4:**
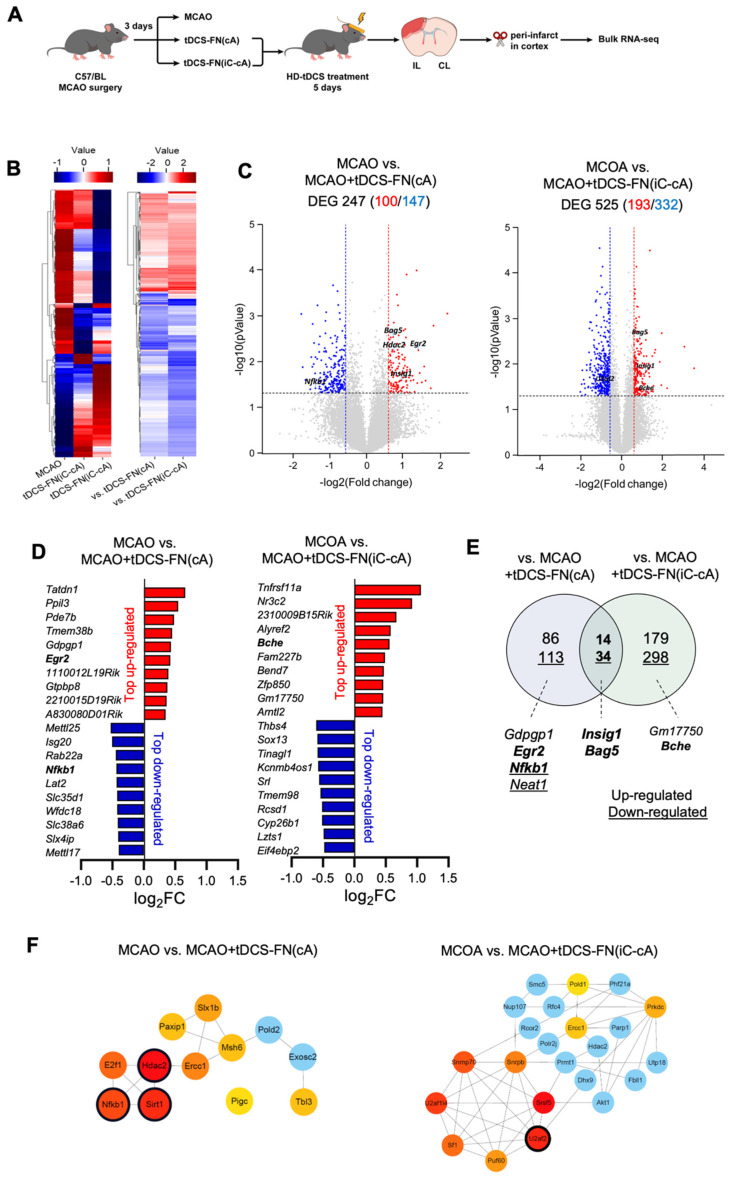
**Bulk RNA-seq transcriptome analysis of the new HD-tDCS application according to electrode configuration. (A)** Experimental timeline and scheme. The cortical peri-infarct tissues were used for bulk RNA-seq (n=3/group). **(B)** Heatmap showing the normalized scaled expression of differentially expressed genes in each and comparison groups. Up-regulation (red) or down-regulation (blue) of genes. **(C)** Volcano plots present the gene expression pattern of the MCAO group compared with that of the MCAO+tDCS-FN(cA) group, as well as the gene expression pattern of the MCAO group compared with that of the MCAO+tDCS-FN(iC-cA) group. **(D)** Bar graph showing the top 10 genes among the up- and downregulated genes between the MCAO and HD-tDCS-FN(cA) groups or HD-tDCS-FN(iC-cA) group. **(E)** Venn diagram of the identified genes for downstream pathway analysis. The up- and downregulated genes are indicated as numbers and underlined numbers, respectively. Comparison of the MCAO and tDCS-FN(cA) groups and that of the MCAO and tDCS-FN(iC-cA) groups, with 14 common upregulated and 34 downregulated genes. **(F)** Verification of hub genes. The top 10 hub genes were selected using MCODE in Cytoscape. MCAO, middle cerebral artery occlusion; HD-tDCS, high-definition transcranial direct current stimulation; FN, new needle-conjugated electrode.

We performed transcriptomic analysis using bulk RNA-seq of the peri-infarct region (**Figure 4A**). We detected 17 417 and 17 444 genes in the MCAO+tDCS-FN(cA) and MCAO+tDCS-FN(iC-cA) groups, respectively, compared with those in the MCAO group. After hierarchical clustering of all groups of DEGs, we compared significantly different gene expression patterns according to electrode configuration by DEGs (**Figure 4B and Data S1**; log_2_ fold change>±1.5; corrected *P*<0.05). We identified 100 upregulated and 147 downregulated genes in the MCAO+tDCS-FN(cA) and MCAO groups, respectively, and 193 upregulated and 332 downregulated genes in the MCAO+tDCS-FN(iC-cA) and MCAO groups, respectively (**Figure 4C**). Of these, the top 10 upregulated and downregulated genes are listed and stroke insult-related genes (early growth response 2 [*Egr2*], nuclear factor kappa B subunit 1 [*Nfkb1*] and butyrylcholinesterase [*Bche*]) are marked in bold in **Figure 4D**. Moreover, up- and downregulated genes were commonly confirmed by both configurations of the HD-tDCS-FN application, including insulin-induced gene 1 (*Insig1*) and bcl-2-associated athanogene 5 (*Bag5*), which are related to stroke insults. We also identified *Egr2* and *Nfkb1* in the vs. MCAO+tDCS-FN(cA) and *Bche* in the vs. MCAO+tDCS-FN(iC-cA) configurations, which are shown in bold in the Venn diagram (**Figure 4E**).

For the network analysis, the hub genes were screened out from the PPI network using the maximal clique centrality algorithm and CytoHubba. The stroke insult-related genes were *Nfkb1*, histone deacetylase 2 (*Hdac2*), and sirtuin 1 (*Sirt1*) in the vs. MCAO+tDCS-FN(cA) and U2 small nuclear RNA auxiliary factor 2 (*U2af2*) in the vs. MCAO+tDCS-FN(iC-cA) configurations (**Figure 4F**). For the GO enrichment analysis of the DEGs, the sterol regulatory element-binding transcription factor (SREBP) and immune response-regulating signaling pathways were the first of the top five up- and downregulated pathways in the biological process, respectively, in vs. MCAO+tDCS-FN(cA) (**Table S1**). Moreover, the KEGG pathway mainly enhanced each specific pathway according to electrode configuration, and common pathways were detected (**Table S1**). These results suggest that both electrode configurations of HD-tDCS manifested specific gene regulation related to stroke insults, and we selected potent genes related to brain injury and protection after stroke, such as *Insig1*, *Bag5*, *Nfkb1*, *Bche*, *Egr2*, *Hdac2*, *Sirt1*, and *U2af2*.

### Potent signaling pathway in therapeutic effects of the new HD-tDCS application

**Figure 5:**
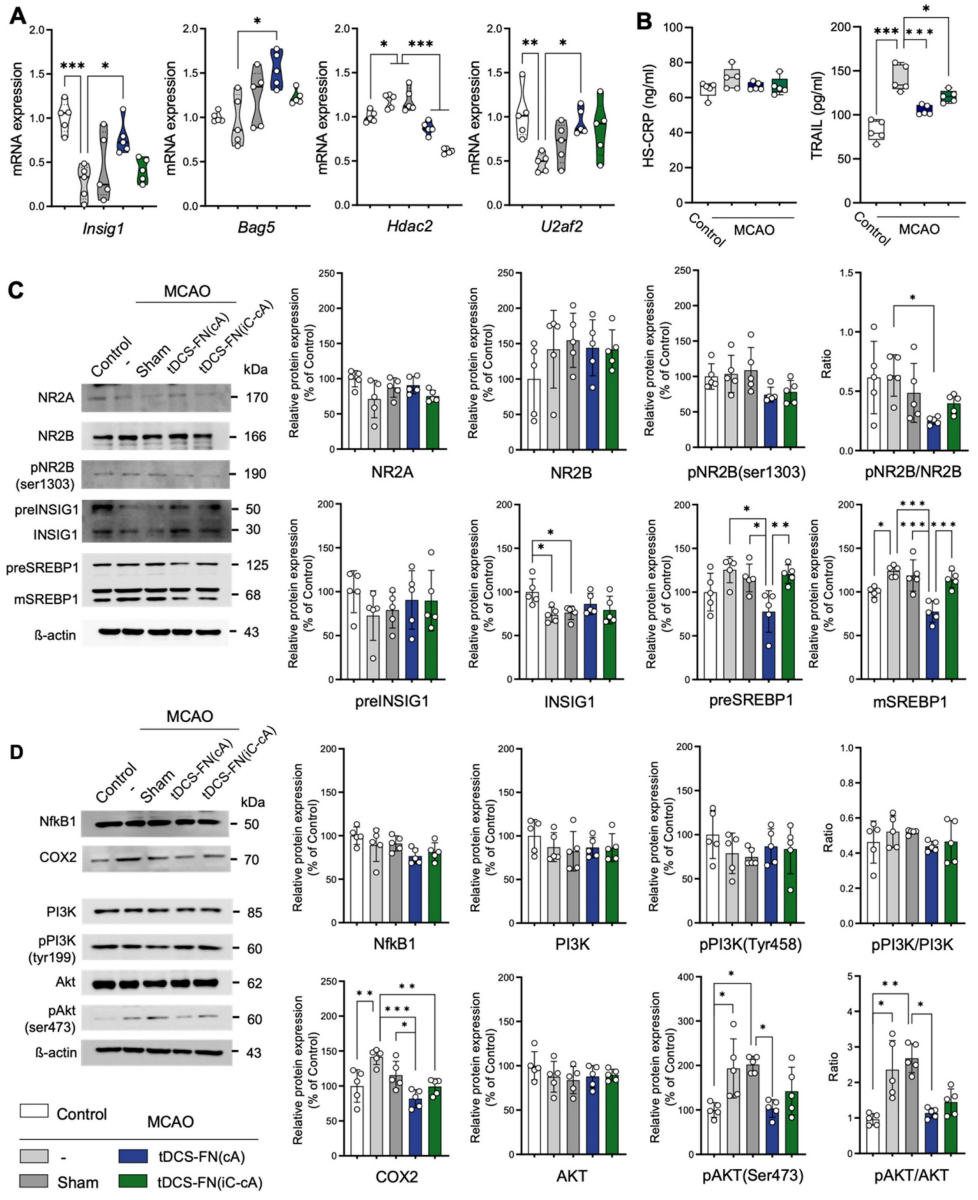
** Verification of potential signaling pathways of the therapeutic effects of the new HD-tDCS application based on transcriptome analysis. (A)** Bar charts of gene expressions for *Insig1*, *Bag5*, *Hdac2*, and *U2af2* in the peri-infarct region using qPCR. The expression of these genes was significantly altered by HD-tDCS-FN application compared to that with MCAO.** (B)** Bar charts of the hs-CRP and TRAIL levels in the peri-infarct region using enzyme-linked immunosorbent assays. The level of TRAIL was significantly reduced in both HD-tDCS-FN groups compared with that in the MCAO group. **(C)** Representative western blots for NMDA-dependent SREBP signaling and related proteins. The pNR2B(ser1303)/NR2B ratio, preSREBP1, and mSREBP1 levels were significantly decreased in the MCAO+tDCS-FN(cA) group compared with those in the MCAO group. **(D)** Representative western blots for inflammation-related proteins. COX2 expression was markedly decreased in both MCAO+tDCS-FN groups compared with that in the MCAO group, while only the pAKT(Ser473)/AKT ratio decreased in the MCAO+tDCS-FN(cA) group. β-actin was used as the loading control. All samples were derived from the peri-infarct region (n=5/group). Data are presented as means±SEMs. *P<0.05, **P<0.01, and ***P<0.001 vs. each group using one-way analysis of variance with Tukey's test. MCAO, middle cerebral artery occlusion; tDCS, transcranial direct current stimulation; FN, new needle-conjugated electrode.

qPCR and western blot analysis were used to verify the potential therapeutic effects of the new HD-tDCS application. In the qPCR analysis, *Insig1*, *Bag5*, *Hdac2*, *Egr2*, and *U2af2,* which are related to cell death pathways, were significantly altered by the new HD-tDCS treatment (**Figure 5A and Figure S2A**). Thus, we analyzed the levels of inflammatory and apoptosis markers, such as hs-CRP and tumor necrosis factor (TNF)-related apoptosis-inducing ligand (TRAIL), using ELISA. The TRAIL levels significantly increased in the MCAO group compared with those in the control group and were significantly reduced in both the HD-tDCS groups (**Figure 5B**).

For the western blot (**Figure S2B**), we focused on the potent proteins involved in *N*-methyl-D-aspartate (NMDA)-dependent SREBP signaling, listed as the first items in the GO enrichment analysis of the DEGs, because *Insig1* is involved in this signaling. The phosphorylated NMDA receptor 2B (NR2B) [pNR2B(ser1303)]/NR2B ratio and pre- and mature SREBP 1 (mSREBP1) expression were significantly reduced in the MCAO+tDCS-FN(cA) group compared with those in the MCAO group, while *Insig1* showed no significant difference (**Figure 5C**). For proteins related to NMDA-dependent inflammatory signaling, both HD-tDCS configurations significantly reduced cyclooxygenase 2 (COX2) expression compared with MCAO, and the phosphorylated AKT [pAKT (ser473)]/ protein kinase B (AKT) ratio was markedly decreased in the MCAO+tDCS-FN(cA) group compared with that in the MCAO group (**Figure 5D**). These results suggest that both HD-tDCS configurations, especially anodal application over the contralesional site, commonly ameliorate the biological signaling of neuronal death via NMDA-dependent SREBP1 signaling and related inflammatory factors, such as COX2 and AKT.

### Immunofluorescence analysis for cell death-related factors in the new HD-tDCS application

**Figure 6:**
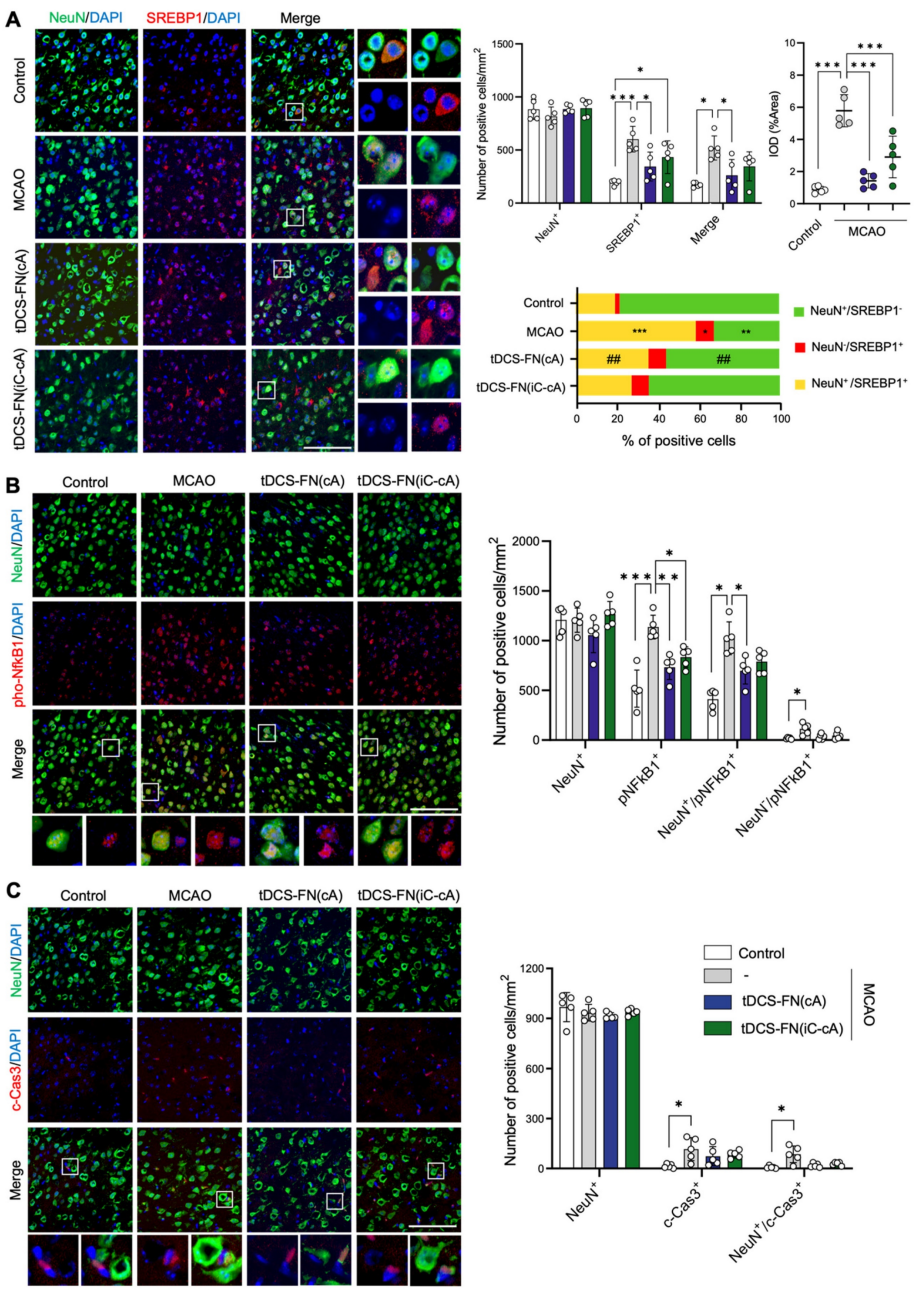
**Immunofluorescence findings for SREBP1 and main inflammatory factors using the new HD-tDCS application. (A)** Photomicrograph and bar chart indicating the number of NeuN- and SREBP1-positive cells, as well as the percentage of the cells. SREBP1-positive and NeuN/SREBP1-double positive cells were significantly reduced by HD-tDCS-FN(cA) application. **(B)** Photomicrograph and bar chart indicating the number of NeuN- and pNFkB1- positive cells. pNFkB1-positive and NeuN/pNFkB1-double positive cells were significantly decreased by HD-tDCS-FN(cA) application. **(C)** Photomicrograph and bar chart showing the number of NeuN- and c-Cas3-positive cells. c-Cas3-positive and NeuN/c-Cas3-double positive cells showed a decreasing tendency with both HD-tDCS-FN treatments. All samples were derived from the peri-infarct region (n=5/group). Data are presented as means±SEMs. *P<0.05, **P<0.01, and ***P<0.001 vs. each group using one-way analysis of variance with Tukey's test. Magnification: ×400; scale bar=100 μm. MCAO, middle cerebral artery occlusion; tDCS, transcranial direct current stimulation; FN, new needle-conjugated electrode.

Finally, we performed immunofluorescence analysis for cell death-related factors, such as SREBP1, NFkB1, and cleaved-caspase3 (c-Cas3), using both configurations of the new HD-tDCS application. Although the number of neuronal nuclei (NeuN)-positive cells did not differ among the groups, the number of SREBP1-positive and NeuN/SREBP1-double positive cells was significantly ameliorated by the application of HD-tDCS-FN(cA) compared to that with MCAO. The IOD of cells in which SREBP1 immunofluorescence overlapped with 4′,6-diamidino-2-phenylindole (DAPI) was measured, indicating the presence of these factors in the nucleus. The number of these cells significantly decreased with the use of both configurations of HD-tDCS compared to that with MCAO. In addition, the percentage of NeuN-positive/SREBP1-negative cells was increased by HD-tDCS-FN(cA) application compared with MCAO, while that of NeuN/SREBP1-double positive cells decreased (**Figure 6A**). The number of phosphorylated NFkB1 (pNFkB1)-positive cells was significantly reduced following the use of both configurations of the new HD-tDCS application compared to that with MCAO, and NeuN/pNFkB1-double cells were significantly decreased only by the HD-tDCS-FN(cA) application (**Figure 6B**). c-Cas3-positive and NeuN/c-Cas3-double positive cells significantly increased in number in the MCAO group compared with those in the control group and were ameliorated by both configurations of the new HD-tDCS application (**Figure 6C**). These results suggest that the HD-tDCS application reduces neuronal cell death in the peri-infarct region after stroke via the regulation of SREBP1 and related inflammation.

## Discussion

We investigated the therapeutic effects of our new HD-tDCS application in early-stage stroke, focusing on motor and cognitive recovery via alleviation of neuronal death in the penumbra. The main findings were that (1) the use of the new HD-tDCS application was associated with a smaller infarct volume with decreased inflammation response after stroke compared with the use of the conventional one and better effects in alleviating motor and cognitive deficits; (2) for the therapeutic effects of the new HD-tDCS application, anodal application over the contralesional site indicated better therapeutic effects than bi-hemispheric stimulation in the early stage of ischemic stroke; and (3) the different electrode configurations of the new HD-tDCS application manifested the regulation of specific genes related to stroke insults, but also commonly ameliorated neuronal death via NMDA-dependent SREBP1 signaling and related inflammatory factors. Collectively, our results support the replacement of the conventional HD-tDCS application for motor and cognitive deficits in patients with neurological disorders.

The interhemispheric competition model, in which an imbalance inhibition occurs between the two hemispheres after a stroke, suggests a possible strategy for tDCS application. Anodal and cathodal tDCS is currently used to upregulate ipsilesional excitability or downregulate contralesional excitability, respectively, or both by simultaneous bi-hemispheric stimulation 2, 3, 29. However, this simplified approach has some limitations because tDCS tools are usually applied to patients with chronic stroke to avoid the potential side effects during the acute phase of stroke 11. Because the degree of ischemic injury might increase throughout the subacute phase, which lasts for a few hours to several days after the stroke onset, treatments may be principally responsible for protecting neurons against apoptosis or necrosis and related inflammatory responses 27, 28. Previous studies suggest that anodal tDCS application at the ipsilesional site induces an increase of the infarct volume via augmentation of blood-brain barrier derangement in the acute phase of stroke, and anodal tDCS treatment of contralesional sites facilitates improvement in behavioral function in the subacute phases of stroke by activating neuronal proliferation and growth factor signaling 11, 26. Therefore, in this study, the conventional and new HD-tDCS anodal electrodes were applied over the contralesional motor area.

tDCS is an effective tool for alleviating motor and cognitive dysfunction following stroke 2, 9, 11. Our results also indicate that the new HD-tDCS application significantly alleviated motor and cognitive deficits and showed better therapeutic effects than the conventional application, as predicted by computational modeling of the electric fields. In particular, reducing infarct size is an important target because it is associated with stroke severity and involves neurological deficits in patients with stroke 30. The new HD-tDCS application significantly reduced the infarct volume and inflammatory response compared with that generated by the conventional application. However, unlike in our previous study 26, conventional HD-tDCS was associated with slight but non-significant functional improvement, which appeared to be due to the timing of treatment after stroke and epicranial stimulation, not due to the stimulation over the scalp. Additionally, the conventional electrode may not have provided sufficient stimulation intensity for cortical excitability.

In the present study, the enhanced target/focus stimulation of the new HD-tDCS based on the electric field was effective in showing better efficacy in the subacute stage of stroke than the conventional one when applied to the contralateral hemisphere. The underlying therapeutic benefits of tDCS are still mostly unknown, but previous studies demonstrate activation of calcium signaling via voltage-gated calcium channels and NMDA receptors 1, 25, 31, 32. Anodal tDCS at the lesional site for early-stage stroke may promote cell death via tDCS-induced overactivation of NMDA glutamate receptors 11, 33. Conversely, the anodal tDCS-induced initial calcium events appear virtually simultaneously in the corresponding contra-anodal regions of the cortex 32. The interhemispheric circuit has a large number of axonal fibers traversing from one hemisphere to the other, and a mouse model of stroke could detect remarkable specificity in alterations in this circuit, highlighting increased reciprocal connectivity 34, 35. Therefore, enhanced focality based on the electric field in contralateral stimulation of new HD-tDCS may affect a reasonable increase of calcium concentrations in the corresponding ipsilateral hemisphere and thereby activate related neuroprotective signaling cascades, such as BDNF 25, 26.

The placement of the electrodes determines the orientation of the electric field; thus, different electrode configurations produce specific neuronal excitability for different clinical outcomes 36, 37. In the early stage of stroke, cathodal tDCS results in better clinical recovery than anodal treatments and correlates with a decrease in infarct volume 11, 38, 39. To determine the therapeutic efficacy of the new HD-tDCS application according to the electrode configuration, we employed two types of configurations: 1) anode HD-tDCS over the contralesional motor cortex and 2) bi-hemispheric stimulation anode HD-tDCS over the contralesional region and cathode over the ipsilesional region. Although both configurations of the new HD-tDCS application alleviated behavioral deficits, such as motor and cognitive deficits in stroke, anodal treatment over the contralesional site showed better therapeutic effects than bi-hemispheric stimulation.

Next, the ischemic penumbra was subjected to RNA-seq analysis between MCAO and the two configurations of the new HD-tDCS applications to identify potential targets of therapeutic effects. In the bioinformatic analysis, each configuration of the new HD-tDCS application revealed regulation of specific genes related to stroke insults, including *Insig1*, *Bag5*, *Nfkb1*, *Bche*, *Egr2*, *Hdac2*, *Sirt1*, and *U2af2*. Since most of these genes are involved in brain injury through cell death after stroke, we examined the apoptotic markers. TRAIL promotes apoptosis following focal brain ischemia by interacting with TRAIL death receptors 40, 41. TRAIL expression was markedly arrested in the HD-tDCS group, suggesting the regulation of cell death.

After verification by qPCR and western blot analysis, we focused on the potential factors of NMDA-dependent SREBP and related inflammatory signaling. Neuronal excitotoxicity caused by overactivation of NMDA glutamate receptors principally contributes to brain injury after cerebral ischemia via the activation of calcium-dependent death signaling 33, 42. The transcription factor SREBP is a major regulator of lipid metabolism and has recently been recognized as a critical transcription factor for excitotoxicity after ischemic stroke 42, 43. NMDA-mediated SREBP1 activation, primarily mediated by the NMDA receptor NR2B subunit, is an essential step in excitotoxic neuronal damage. The activation of the NMDA receptor produces soluble mature *N*-terminal SREBP-1 (68 kDa) for transcriptional activity by increasing *Insig1* degradation 43, 44. Therefore, agents or therapeutic tools that arrest SREBP-1 activation represent a new type of neuroprotective therapy for stroke.

Extrasynaptic activation of the NR2B-containing NMDA receptor leads to excitotoxic neuronal death via various signaling pathways, including those involving calpain, STEP, p38, JNK, and SREBP1, which are coupled further downstream. However, synaptic activation of the *N*‑methyl‑D‑aspartate receptor 2A (NR2A)-containing NMDA receptor triggers pro-survival (Akt, ERK, and CREB) signaling 42. Our results suggest that the new HD-tDCS application may ameliorate neuronal death in the penumbra after stroke via the regulation of the NMDA receptor NR2B subunit and further trigger the activation of the SREBP1 signaling pathway. In addition, potential mediators of inflammation and cell death such as COX are involved in the underlying therapeutic effects. SREBP1 is mainly distributed in neurons 45, and our immunofluorescence findings confirmed that the therapeutic outcomes of the new HD-tDCS application were mediated via the inhibition of neuronal apoptosis by SREBP1 and inflammatory factors. Previous evidence suggests that tDCS increases cortical excitability in an NMDA receptor-dependent manner and is linked to downstream molecular cascades for long-lasting after-effects via changes in intracellular calcium levels 25, 31, 32. Therefore, the new HD-tDCS application may exert a neuroprotective effect in the early stages of ischemic stroke through the regulation of NMDA receptor-linked downstream molecular cascades.

However, transcranial electrical stimulation alters the blood flow in the brain as well as neuronal stimulation, which reduces the volume of the infarct region to change the inflammatory response in ischemic stroke 46-48. Distant interhemispheric projections also evoke neurovascular responses 34. It is possible that global CBF changes related to higher density and focality of the HD-tDCS stimulation in ischemic stroke may have a neuroprotective function.

Our study highlights several points to consider for bench-to-bedside translation. The needle-conjugated HD electrode allows more targeted and effective stimulation of a specific brain cortex by its positioning near the sites; however, the use of a needle requires minimal invasion of the scalp because of possible infection. Current spreads more evenly in conventional flat HD electrodes; however, our new HD electrode is likely to concentrate the current at sharp geometric gradients at the tip of the needle. Although no histological injuries to the scalp were observed in this study, they cause uncomfortable sensations such as edge effects at the corners of large rectangular electrodes 16, 22, 49. The translational value of the new HD-tDCS in patients with early-stage stroke may be limited by invasive skin injuries, but scalp acupuncture is also employed for such patients, suggesting that the new HD-tDCS might be feasible 50. In addition, long-term evaluation will be necessary for post-stroke research because patients with stroke usually reach a recovery plateau following the subacute period. Considering the aforementioned points, further clinical trials are required to review and to improve on the problems that can result from the use of the new HD electrode.

Although the exact mechanism of HD-tDCS in modulating brain function after stroke is not fully known, our study supports the use of this HD-tDCS application as an effective therapy for motor and cognitive deficits via a neuroprotective effect in patients with early-stage stroke, and it might also apply to brain injuries due to inflammation and cell death. Moreover, our new HD electrode allows for more targeted and effective stimulation of specific regions of the cortex in other brain disorders and ensures consistent placement with minimal movement of the electrode during the procedure.

## Supplementary Material

Supplementary figures and table.
