# Supplementary material for: Therapeutic effects of a novel electrode for transcranial direct current stimulation in ischemic stroke mice
Source: Theranostics. 2024 Jan 21;14(4):1325–43. doi: 10.7150/thno.90779 (PMC10879864; doi:10.7150/thno.90779)
Supplement: Supplementary file 1 — Supplementary figures and table. [file thnov14p1325s1.pdf]

Supplementary Information

Therapeutic effects of a novel electrode for transcranial direct current stimulation in ischemic stroke mice

Da Hee Jung[a,b], *Jae Ho Lee*[a,b], *Hong Ju Lee*[a], *Jang Woo Park*[c], *Young-Jin Jung*[d], *Hwa Kyoung Shin*[a,b,*], *Byung Tae Choi*[a,b,*]

[a]Department of Korean Medical Science, School of Korean Medicine, Pusan National University, Yangsan 50612, Republic of Korea

[b]Graduate Training Program of Korean Medical Therapeutics for Healthy Aging, Pusan National University, Yangsan 50612, Republic of Korea

[c]Korea Radioisotope Center for Pharmaceuticals, Korea Institute of Radiological & Medical Sciences, Seoul 01812, Republic of Korea

[d]School of Healthcare and Biomedical Engineering, Chonnam National University, Yeosu 59626, Republic of Korea

[*]**Corresponding authors**: Byung Tae Choi and Hwa Kyoung Shin, Department of Korean Medical Science, School of Korean Medicine, Pusan National University, Yangsan 50612, Republic of Korea

Telephone: +82-51-510-8475; Fax: +82-51-510-8437

E-mail: choibt@pusan.ac.kr, julie@pusan.ac.kr

This PDF file includes:

Figures S1- S2
Table S1

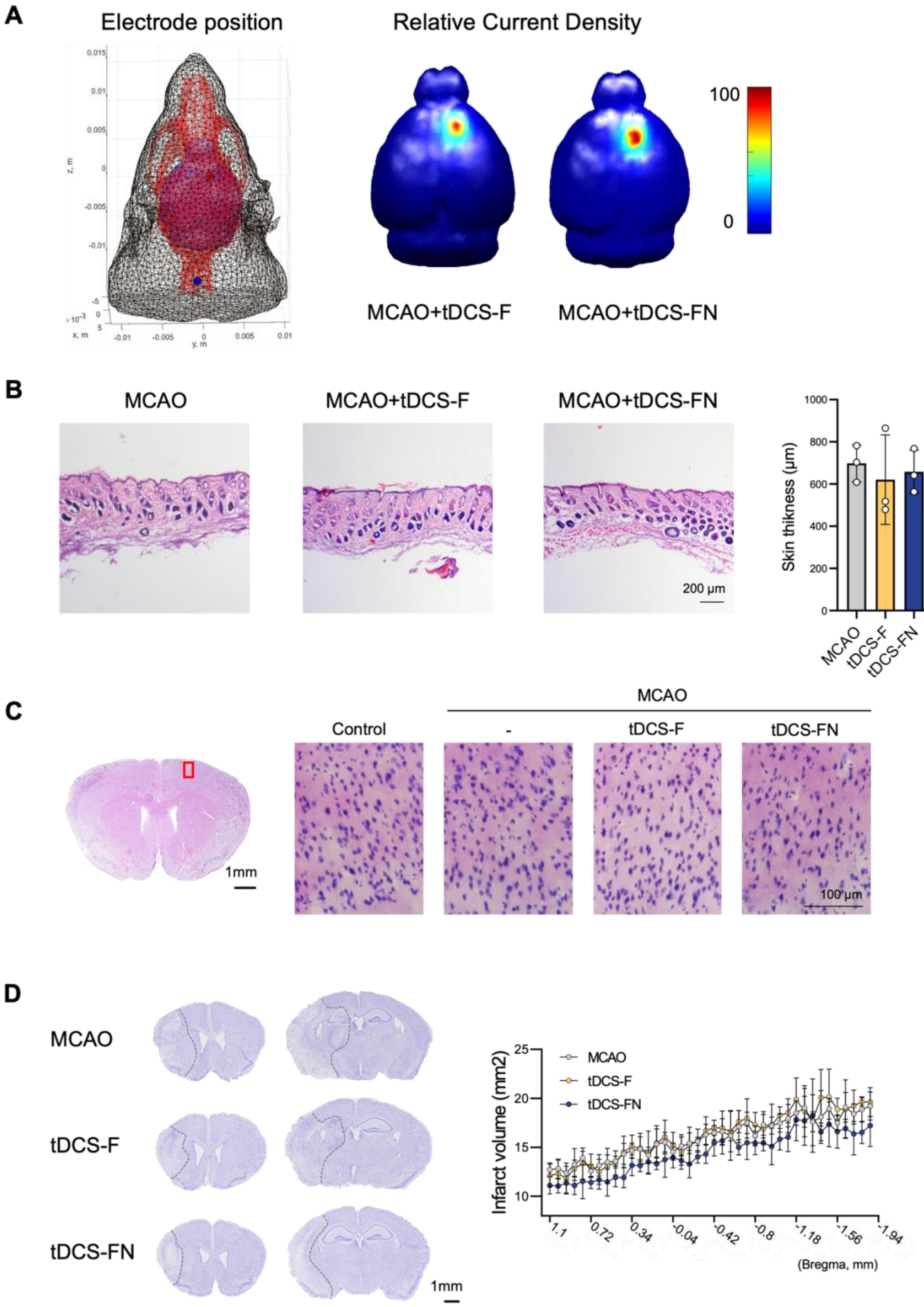
A
Electrode position
Relative Current Density
100
0
MCAO+tDCS-F
MCAO+tDCS-FN
B
MCAO
MCAO+tDCS-F
MCAO+tDCS-FN
200 μm
Skin thikness (μm)
MCAO
tDCS-F
tDCS-FN
C
MCAO
Control
-
tDCS-F
tDCS-FN
1mm
100 μm
D
MCAO
tDCS-F
tDCS-FN
1mm
Infarct volume (mm2)
(Bregma, mm)

Figure Sl. The 3D simulation of HD-tDCS and histological examination of the scalp. (A) 3D simulation of HD-tDCS. Higher relative electrical fields and spatial focality were predicted in the motor cortex using the new HD electrode compared with the conventional HD electrode. **(B)** Histological examination and bar chart of the scalp by hematoxylin–eosin (H&E) staining. No pathological injuries were observed in the HD-tDCS-treated groups compared with the non-treated group (n=3/group). (**C**) Histological examination of the cerebral cortex by H&E staining. There were no pathological injuries by conventional or new HD electrode stimulation. (**D**) Representative brain images (0.98 mm and -1.58 mm from bregma) and quantification of the relative infarct volume. Borders to the healthy tissue are indicated with a dot line. Nissl staining: In total, 40 brain sections were identified every 75 μm at 1.1 mm to −1.9 mm from the bregma (n=5/group). Data are presented as means±SEMs. Magnification: ×25; scale bar=200 μm. HD-tDCS, high-definition transcranial direct current stimulation; MCAO, middle cerebral artery occlusion. F, conventional electrode; FN, new needle-conjugated electrode.

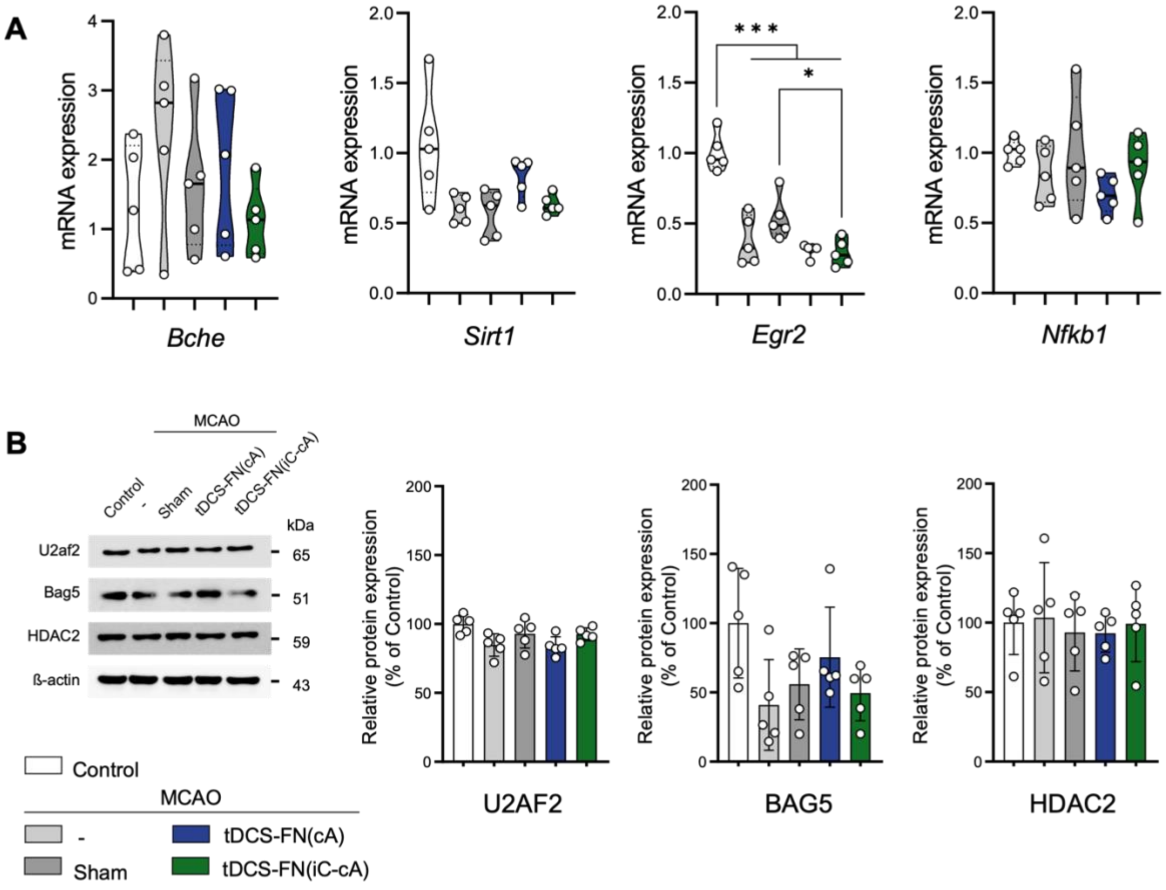

Figure S2. Verification of the potential signaling pathway of the therapeutic effects of HD-tDCS using the newly-designed electrode, based on the transcriptome analysis. (A) Bar charts of the gene expressions of *Bche*, *Sirt1* and *Nfkb1*, and *Egr2* in the peri-infarct region using qPCR. **(B)** Representative western blots for U2AF2, BAG5, and HDAC2. In all groups, no significant changes were induced in gene or protein expression. All samples were obtained from the peri-infarct region (n=5/group). tDCS, transcranial direct current stimulation; FN, new needle-conjugated electrode.

Table S1. GO and KEGG pathways according to the electrode configuration of the new HD-tDCS application.

List		Count	%	-log (Pvalue)	Genes	Fold Enrichment
vs. tDCS-FN(cA)	**UP regulation genes in DEG**					
	**GO_Biological process**					
	GO:0032933~SREBP signaling pathway	2	2.11	1.38	*INSIG1*, *EIF2A*	46.25
	GO:1903351~cellular response to dopamine	2	2.11	1.34	*HDAC2*, *GABPA*	42.39
	GO:2000736~regulation of stem cell differentiation	2	2.11	1.13	*HDAC2*, *SMYD5*	25.44
	GO:0045737~positive regulation of cyclin-dependent protein serine/threonine kinase activity	2	2.11	1.03	*CDK5RAP1*, *MNAT1*	20.35
	GO:0016575~histone deacetylation	3	3.16	1.78	*HDAC2*, *BCL6*, *MSL3*	14.96
	GO:0006260~DNA replication	3	3.16	1.09	*RRM2B*, *POLD2*, *DONSON*	6.25
	GO:0006281~DNA repair	6	6.32	1.77	*MSH6*, *DCLRE1A*, *RRM2B*, *POLD2*, *MNAT1*, *RNF111*	3.97
	GO:0006974~cellular response to DNA damage stimulus	6	6.32	1.32	*MSH6*, *DCLRE1A*, *BCL6*, *RRM2B*, *POLD2*, *RNF111*	3.00
	GO:0006357~regulation of transcription from RNA polymerase II promoter	15	15.79	2.48	*ZFP799*, *HDAC2*, *EGR2*, *ZFP935*, *ZFP438*, *ZFP426*, *ZBTB2*, *MED23*, *BCL6*, *ZFP40*, *MNAT1*, *ZFP212*, *ZFP300*, *ZFP9*, *GABPA*	2.38
	**GO_Cellular component**					
	GO:0005657~replication fork	3	3.16	2.29	*HDAC2*, *BCL6*, *DONSON*	27.73
	GO:0016234~inclusion body	2	2.11	1.07	*PSMC6*, *BAG5*	22.34
	GO:0005654~nucleoplasm	28	29.47	4.23	*DCLRE1A*, *HDAC2*, *IPO11*, *GTF3C5*, *SPATA7*, *ZBTB2*, *COIL*, *TATDN1*, *MKNK2*, *POLD2*, *POLR2H*, *EXOSC2*, *RNF111*, *GABPA*, *EGR2*, *ZFP438*, *PLEKHA5*, *TTI2*, *MED23*, *MSH6*, *TBL3*, *RRM2B*, *BCL6*, *DNAJC7*, *NUP98*, *SRSF5*, *MNAT1*, *ZFP212*	2.18
	GO:0005794~Golgi apparatus	10	10.53	1.11	*MSH6*, *SLC35A1*, *RAB30*, *PRRC1*, *RAB40B*, *BCL6*, *ATL1*, *COPG2*, *GBA2*, *CLN5*	1.88
	GO:0005634~nucleus	35	36.84	2.05	*DCLRE1A*, *HDAC2*, *IPO11*, *GTF3C5*, *ZFP426*, *COIL*, *TATDN1*, *BAG5*, *RAVER1*, *MKNK2*, *POLD2*, *POLR2H*, *ZFP300*, *EXOSC2*, *RPS10*, *RNF111*, *GABPA*, *ZFP799*, *EGR2*, *ZFP438*, *DONSON*, *MSL3*, *MED23*, *MSH6*, *TBL3*, *PSMC6*, *RRM2B*, *BCL6*, *DNAJC7*, *ZFP40*, *AKT1S1*, *NUP98*, *SRSF5*, *MNAT1*, *BRDT*	1.46
	GO:0005829~cytosol	22	23.16	1.10	*IPO11*, *PIGN*, *ZFP438*, *PLEKHA5*, *MYO19*, *GBA2*, *SPATA7*, *TTI2*, *CLN5*, *MSH6*, *PEX7*, *BAG5*, *AP5M1*, *DNAJC7*, *RRM2B*, *AKT1S1*, *CDK5RAP1*, *COPG2*, *SRSF5*, *EXOSC2*, *RPS10*, *RNF111*	1.41
	**GO_Molecular function**					

GO:0031072~heat shock protein binding	3	3.16	1.39	*HDAC2, BAG5, DNAJC7*	9.23
GO:0003729~mRNA binding	4	4.21	1.02	*RAVER1, SRSF5, NUP98, EIF2A*	3.62
GO:0000978~RNA polymerase II core promoter proximal region sequence-specific DNA binding	10	10.53	1.36	*EGR2, HDAC2, BCL6, ZFP935, ZFP40, ZFP426, ZFP212, ZFP300, GABPA, ZBTB2*	2.11
GO:0016787~hydrolase activity	13	13.68	1.59	*DCLRE1A, HDAC2, ENTPD1, ATL1, PTPRO, GBA2, NT5C3B, TATDN1, PDE7B, NUP98, MEST, GDPGP1, TRABD2B*	1.99
GO:0000166~nucleotide binding	13	13.68	1.40	*ENTPD1, MARS2, ATL1, GTPBP8, MYO19, MSH6, NT5C3B, RAB30, PSMC6, RAB40B, MKNK2, MAP3K6, GDPGP1*	1.86
GO:0046872~metal ion binding	24	25.26	1.96	*ZFP799, DCLRE1A, HDAC2, EGR2, ZFP935, SMYD5, ZFP426, GTPBP8, NT5C3B, TATDN1, RRM2B, BCL6, ZFP40, ZFYVE19, MKNK2, CDK5RAP1, PDE7B, MAP3K6, MNAT1, ZFP212, ZFP300, ZFP9, TRABD2B, RNF111*	1.65
**KEGG pathway**					
mmu03430:Mismatch repair	2	2.11	1.07	*MSH6, POLD2*	22.09
mmu00240:Pyrimidine metabolism	3	3.16	1.68	*NT5C3B, ENTPD1, RRM2B*	13.02
mmu01232:Nucleotide metabolism	3	3.16	1.35	*NT5C3B, ENTPD1, RRM2B*	8.68
mmu05168:Herpes simplex virus 1 infection	8	8.42	2.70	*ZFP799, ZFP935, ZFP40, ZFP426, SRSF5, ZFP212, ZFP300, ZFP9*	4.24
**Down regulation genes in DEG**					
**GO_Biological process**					
GO:0002764~immune response-regulating signaling pathway	2	1.41	1.90	*LAT2, CTSH*	158.22
GO:1904431~positive regulation of t-circle formation	2	1.41	1.73	*SLX1B, ERCC1*	105.48
GO:1990410~adrenomedullin receptor signaling pathway	2	1.41	1.60	*RAMP3, ADM*	79.11
GO:0000720~pyrimidine dimer repair by nucleotide-excision repair	2	1.41	1.60	*ERCC1, SIRT1*	79.11
GO:0090656~t-circle formation	2	1.41	1.51	*SLX1B, ERCC1*	63.29
GO:0031102~neuron projection regeneration	2	1.41	1.51	*ULK1, ADM*	63.29
GO:0071322~cellular response to carbohydrate stimulus	2	1.41	1.43	*RPS6KA2, NFKB1*	52.74
GO:0035166~post-embryonic hemopoiesis	2	1.41	1.43	*MYO1E, ERCC1*	52.74
GO:0097647~amylin receptor signaling pathway	2	1.41	1.37	*RAMP3, ADM*	45.21
GO:0001936~regulation of endothelial cell proliferation	2	1.41	1.26	*KDR, TEK*	35.16
**GO_Cellular component**					
GO:0000506~glycosylphosphatidylinositol-N-acetylglucosaminyltransferase (GPI-GnT) complex	2	1.41	1.33	*PIGC, PIGA*	41.37
GO:0031201~SNARE complex	3	2.11	1.46	*STX2, SYN2, VAMP3*	10.13
GO:0005902~microvillus	3	2.11	1.00	*MYO1E, PDGFA, TEK*	5.58

GO:0008021~synaptic vesicle	5	3.52	1.62	*GRIN2A, PSEN2, HAP1, STX2, SYN2*	4.55
GO:0005769~early endosome	9	6.34	3.05	*PSEN2, KDR, AP1S3, ACKR1, HAP1, TM9SF4, RAB22A, FGD2, VAMP3*	4.49
GO:0005765~lysosomal membrane	5	3.52	1.14	*ABCA2, LAPTM5, PSEN2, AP1S3, WDR24*	3.16
GO:0005764~lysosome	8	5.63	1.67	*ABCA2, RAMP3, LAPTM5, CTSH, ARSG, HAP1, WDR24, SGSH*	2.87
GO:0031410~cytoplasmic vesicle	13	9.15	2.18	*ABCA2, PANK1, WFS1, LAPTM5, RAB22A, MYO1E, GRIN2A, KDR, AP1S3, HAP1, ADGRL4, STX2, VAMP3*	2.45
GO:0030054~cell junction	12	8.45	1.57	*CAMSAP3, MYO1E, GRIN2A, MAST2, NPHP1, KDR, TEK, HAP1, SYN2, GABRG1, SNTA1, VAMP3*	2.10
GO:0009986~cell surface	9	6.34	1.03	*GRIN2A, RAMP3, PSEN2, KDR, PDGFA, TEK, STX2, VAMP5, VAMP3*	1.93
**GO_Molecular function**					
GO:0017176~phosphatidylinositol N-acetylglucosaminyltransferase activity	2	1.41	1.38	*PIGC, PIGA*	46.90
GO:0008484~sulfuric ester hydrolase activity	2	1.41	1.12	*ARSG, SGSH*	25.25
GO:0004521~endoribonuclease activity	3	2.11	1.72	*ZC3H12C, YBEY, LACTB2*	14.07
GO:0004518~nuclease activity	6	4.23	2.91	*ISG20, ZC3H12C, YBEY, SLX1B, LACTB2, ERCC1*	7.46
GO:0004519~endonuclease activity	4	2.82	1.70	*ZC3H12C, YBEY, SLX1B, LACTB2*	6.91
GO:0008168~methyltransferase activity	5	3.52	1.62	*METTL25, PRDM4, METTL8, TRMT61A, METTL17*	4.53
GO:0005516~calmodulin binding	5	3.52	1.50	*CAMSAP3, MYO1E, WFS1, PHKA2, SNTA1*	4.17
GO:0016740~transferase activity	23	16.20	3.10	*PRDM4, PANK1, MAST2, NSMCE2, IBA57, DTX4, PIGV, FHIT, SIRT1, PAFAH2, TRIT1, PAPSS1, METTL25, PIGA, RPS6KA2, KDR, ULK1, IDNK, TEK, METTL8, TRMT61A, METTL17, TGM2*	2.14
GO:0016787~hydrolase activity	18	12.68	1.79	*USP36, HDDC2, SLX1B, PSEN2, DUSP18, HEXDC, FHIT, PAFAH2, ISG20, ZC3H12C, YBEY, LACTB2, HMCES, AFMID, CTSH, ARSG, HIBCH, TGM2*	1.84
GO:0000166~nucleotide binding	16	11.27	1.03	*ABCA2, PANK1, MAST2, FHIT, GTPBP1, RAB22A, TRIT1, PAPSS1, MYO1E, RPS6KA2, KDR, ULK1, IDNK, TTLL12, TEK, TGM2*	1.53
**KEGG pathway**					
mmu00563:Glycosylphosphatidylinositol (GPI)-anchor biosynthesis	3	2.11	1.83	*PIGC, PIGA, PIGV*	15.72
mmu04130:SNARE interactions in vesicular transport	3	2.11	1.63	*STX2, VAMP5, VAMP3*	12.39
mmu04142:Lysosome	6	4.23	2.55	*ABCA2, LAPTM5, CTSH, AP1S3, ARSG, SGSH*	6.06
mmu04722:Neurotrophin signaling pathway	5	3.52	1.95	*PRDM4, RPS6KA2, ARHGDIA, PSEN2, NFKB1*	5.63
mmu00230:Purine metabolism	4	2.82	1.14	*HDDC2, PGM2, FHIT, PAPSS1*	4.07
mmu04015:Rap1 signaling pathway	5	3.52	1.16	*GRIN2A, KDR, PDGFA, TEK, SIPA1L2*	3.18
mmu04014:Ras signaling pathway	5	3.52	1.05	*GRIN2A, KDR, PDGFA, TEK, NFKB1*	2.90

	mmu01100:Metabolic pathways	19	13.38	1.45	*HDDC2, GLDC, PANK1, HEXDC, PIGV, FHIT, SIRT1, PAFAH2, TRIT1, PAPSS1, SGSH, PIGC, ALDH3B1, PIGA, AFMID, PGM2, IDNK, L2HGDH, HIBCH*	1.60
vs. tDCS-FN(iC-cA)	**<u>UP regulation genes in DEG</u>**					
	**GO_Biological process**					
	GO:0006850~mitochondrial pyruvate transport	2	1.08	1.78	*MPC1, MPC2*	119.61
	GO:0098942~retrograde trans-synaptic signaling by trans-synaptic protein complex	2	1.08	1.39	*FARP1, NLGN1*	47.84
	GO:0097114~NMDA glutamate receptor clustering	2	1.08	1.31	*NLGN1, HTR1A*	39.87
	GO:0060527~prostate epithelial cord arborization involved in prostate glandular acinus morphogenesis	2	1.08	1.25	*FRS2, IGF1*	34.17
	GO:1903608~protein localization to cytoplasmic stress granule	2	1.08	1.19	*DHX9, DCP1A*	29.90
	GO:0061732~mitochondrial acetyl-CoA biosynthetic process from pyruvate	2	1.08	1.19	*MPC1, MPC2*	29.90
	GO:2000726~negative regulation of cardiac muscle cell differentiation	2	1.08	1.19	*HDAC3, FRS2*	29.90
	GO:0006625~protein targeting to peroxisome	2	1.08	1.10	*ZFAND6, LONP2*	23.92
	GO:0046620~regulation of organ growth	2	1.08	1.10	*LATS1, SAV1*	23.92
	GO:0009312~oligosaccharide biosynthetic process	2	1.08	1.06	*MGAT2, ST6GALNAC6*	21.75
	**GO_Cellular component**					
	GO:0072487~MSL complex	2	1.08	1.41	*MSL3, MSL3L2*	50.43
	GO:0005641~nuclear envelope lumen	2	1.08	1.01	*BCHE, CACYBP*	19.39
	GO:0000118~histone deacetylase complex	3	1.62	1.33	*HDAC2, HDAC3, RCOR2*	8.60
	GO:0032391~photoreceptor connecting cilium	3	1.62	1.29	*CETN2, CEP290, SPATA7*	8.22
	GO:0022627~cytosolic small ribosomal subunit	3	1.62	1.24	*RPS27, RPS21, RPS10*	7.72
	GO:0019005~SCF ubiquitin ligase complex	4	2.16	1.76	*FBXW11, CACYBP, FBXO32, FBXO33*	7.31
	GO:0022626~cytosolic ribosome	4	2.16	1.58	*RPS27, RPS21, RPL9, RPS10*	6.23
	GO:0005762~mitochondrial large ribosomal subunit	3	1.62	1.07	*MRPL20, MRPL1, MIEF1*	6.10
	GO:0017053~transcriptional repressor complex	3	1.62	1.04	*HDAC2, HDAC3, C1D*	5.91
	GO:0098982~GABA-ergic synapse	5	2.70	1.96	*NLGN1, RPS27, GLRB, HTR1A, NR3C2*	5.78
	**GO_Molecular function**					
	GO:0046972~histone acetyltransferase activity (H4-K16 specific)	2	1.08	1.49	*MSL3, MSL3L2*	60.76
	GO:0050833~pyruvate transmembrane transporter activity	2	1.08	1.49	*MPC1, MPC2*	60.76
	GO:0001069~regulatory region RNA binding	2	1.08	1.40	*DHX9, IREB2*	48.61
	GO:1902388~ceramide 1-phosphate transporter activity	2	1.08	1.25	*CPTP, MTTP*	34.72
	GO:0005548~phospholipid transporter activity	2	1.08	1.15	*PITPNM1, MTTP*	27.00

GO:0032041~NAD-dependent histone deacetylase activity (H3-K14 specific)	2	1.08	1.10	*HDAC2, HDAC3*	24.30
GO:0033558~protein deacetylase activity	2	1.08	1.03	*HDAC2, HDAC3*	20.25
GO:0005212~structural constituent of eye lens	4	2.16	2.92	*CRYGS, CRYBA2, CRYBB2, CRYBA4*	18.69
GO:0051059~NF-kappaB binding	4	2.16	2.65	*HDAC2, HDAC3, FAF1, COMMD6*	15.19
GO:0031072~heat shock protein binding	6	3.24	3.30	*HDAC2, PACRG, BAG5, GPR37, DNAJC7, FAF1*	9.11
**KEGG pathway**					
mmu03420:Nucleotide excision repair	3	1.62	1.22	*RFC4, CETN2, POLD1*	7.38
mmu03010:Ribosome	6	3.24	1.59	*MRPL20, MRPL1, RPS27, RPS21, RPL9, RPS10*	3.55
**Down regulation genes in DEG**					
**GO_Biological process**					
GO:0031587~positive regulation of inositol 1,4,5-trisphosphate-sensitive calcium-release channel activity	2	0.64	1.26	*HTT, HAP1*	35.88
GO:0018193~peptidyl-amino acid modification	2	0.64	1.26	*ASPHD1, ASPHD2*	35.88
GO:0051386~regulation of neurotrophin TRK receptor signaling pathway	2	0.64	1.26	*DOK5, ULK1*	35.88
GO:0001998~angiotensin-mediated vasoconstriction involved in regulation of systemic arterial blood pressure	2	0.64	1.26	*G6PDX, AGT*	35.88
GO:2001170~negative regulation of ATP biosynthetic process	2	0.64	1.26	*FLCN, PARP1*	35.88
GO:0071205~protein localization to juxtaparanode region of axon	2	0.64	1.17	*NFASC, CNTN2*	28.71
GO:0032929~negative regulation of superoxide anion generation	2	0.64	1.17	*PON3, AKT1*	28.71
GO:0014873~response to muscle activity involved in regulation of muscle adaptation	2	0.64	1.17	*SRL, AGT*	28.71
GO:0099504~synaptic vesicle cycle	2	0.64	1.17	*SYN2, SYN1*	28.71
GO:0048858~cell projection morphogenesis	2	0.64	1.09	*CAMK2B, CSPG5*	23.92
**GO_Cellular component**					
GO:0089701~U2AF	3	0.96	2.45	*U2AF2, U2AF1L4, SF1*	31.96
GO:0098984~neuron to neuron synapse	2	0.64	1.19	*ATP1A3, ATP1B2*	29.83
GO:0060203~clathrin-sculpted glutamate transport vesicle membrane	2	0.64	1.05	*OTOF, SYN1*	21.31
GO:0033018~sarcoplasmic reticulum lumen	2	0.64	1.05	*CALU, SRL*	21.31
GO:0071004~U2-type prespliceosome	3	0.96	1.72	*U2AF2, SNRNP70, SNRPB*	13.98
GO:0005774~vacuolar membrane	4	1.29	2.41	*NAPA, SLC22A17, ATP6V0C, WDR24*	12.43
GO:0048787~presynaptic active zone membrane	3	0.96	1.63	*OTOF, APBA1, STX2*	12.43
GO:0033268~node of Ranvier	3	0.96	1.54	*NFASC, KCNQ2, CNTN2*	11.19
GO:0035371~microtubule plus-end	3	0.96	1.43	*DCTN1, CLIP4, SLAIN1*	9.73
GO:0030285~integral component of synaptic vesicle membrane	6	1.93	3.36	*SYNGR1, SLC6A7, ATP6V1G2, SLC30A3, SV2A, VAMP3*	9.32

**GO_Molecular function**					
GO:0033897~ribonuclease T2 activity	2	0.64	1.56	*RNASET2B, RNASET2A*	72.53
GO:0004376~glycolipid mannosyltransferase activity	2	0.64	1.39	*PIGB, PIGV*	48.35
GO:0047179~platelet-activating factor acetyltransferase activity	2	0.64	1.27	*PAFAH1B3, PAFAH2*	36.27
GO:0003847~1-alkyl-2-acetylglycerophosphocholine esterase activity	3	0.96	2.30	*PLA2G6, PAFAH1B3, PAFAH2*	27.20
GO:0030628~pre-mRNA 3'-splice site binding	2	0.64	1.03	*U2AF2, U2AF1L4*	20.72
GO:0004708~MAP kinase kinase activity	3	0.96	1.70	*MAP2K3, MAP2K2, MAP2K7*	13.60
GO:0004712~protein serine/threonine/tyrosine kinase activity	5	1.61	3.20	*PRKCG, CLK1, MAP2K2, AKT1, DYRK1B*	12.51
GO:0004521~endoribonuclease activity	5	1.61	2.89	*RCL1, YBEY, AGO2, RNASET2B, RNASET2A*	10.36
GO:0042054~histone methyltransferase activity	3	0.96	1.48	*SUV39H1, CARM1, SMYD1*	10.36
GO:0016409~palmitoyltransferase activity	3	0.96	1.37	*ZDHHC12, ZDHHC6, ZDHHC4*	9.07
**KEGG pathway**					
mmu00563:Glycosylphosphatidylinositol (GPI)-anchor biosynthesis	3	0.96	1.28	*PIGB, GPAA1, PIGV*	8.04
mmu04721:Synaptic vesicle cycle	6	1.93	2.33	*NAPA, SLC6A7, ATP6V1G2, STX2, ATP6V0C, CPLX1*	5.43
mmu04961:Endocrine and other factor-regulated calcium reabsorption	4	1.29	1.25	*PRKCG, ATP1A3, ATP1B2, SLC8A2*	4.57
mmu04664:Fc epsilon RI signaling pathway	4	1.29	1.17	*MAP2K3, MAP2K2, AKT1, MAP2K7*	4.22
mmu04012:ErbB signaling pathway	5	1.61	1.50	*PRKCG, CAMK2B, MAP2K2, AKT1, MAP2K7*	4.15
mmu04261:Adrenergic signaling in cardiomyocytes	9	2.89	2.84	*CAMK2B, CACNB1, TPM2, AKT1, ATP1A3, ATP1B2, ADRA1B, AGT, SLC8A2*	4.13
mmu04722:Neurotrophin signaling pathway	7	2.25	2.12	*CAMK2B, MAPK7, MAP2K2, PRDM4, PSEN2, AKT1, MAP2K7*	4.03
mmu04260:Cardiac muscle contraction	5	1.61	1.45	*CACNB1, TPM2, ATP1A3, ATP1B2, SLC8A2*	4.01
mmu04912:GnRH signaling pathway	5	1.61	1.41	*CAMK2B, MAP2K3, MAPK7, MAP2K2, MAP2K7*	3.87
mmu04918:Thyroid hormone synthesis	4	1.29	1.05	*PRKCG, ATP1A3, ATP1B2, TTF1*	3.77
